# A Rapid Assessment Approach for Skin Stratum‐Targeted Drug Delivery Systems Using Mass Spectrometry Imaging and Spatial Clustering

**DOI:** 10.1002/smsc.202500061

**Published:** 2025-06-01

**Authors:** Ravit Yakobi Arancibia, Einav Bentov‐Arava, Anna Morshin, Jhonathan Elia, Hiba Natsheh, Yael Levi‐Kalisman, Rotem Ushki, Anna Elia, Elka Touitou, Katherine Margulis

**Affiliations:** ^1^ The Institute for Drug Research, the School of Pharmacy Faculty of Medicine The Center for Nanoscience and Nanotechnology The Hebrew University of Jerusalem Jerusalem 9112001 Israel; ^2^ Department of Plastic Surgery The Hadassah Medical Center Hebrew University School of Medicine POB 12000 Jerusalem il‐91120 Israel; ^3^ Institute of Life Sciences and the Center for Nanoscience and Nanotechnology The Hebrew University of Jerusalem Edmond Safra Campus Givat Ram Jerusalem 9190401 Israel; ^4^ Department of Pathology Faculty of Medicine Hadassah Medical Center The Hebrew University of Jerusalem Jerusalem 91120 Israel

**Keywords:** dermal delivery, desorption electrospray ionization mass spectrometry imaging, ethosomes, microemulsion, skin layers, terbinafine, transethosomes

## Abstract

A novel mass spectrometry imaging (MSI)‐based concept that enables rapid visualization and evaluation of active pharmaceutical ingredient (API) distribution across skin layers following dermal delivery is presented. This approach integrates desorption electrospray ionization MSI with a newly developed automated computational tool (access provided) that efficiently processes MSI data, isolates skin tissue signals from background interference, and segments the tissue into precise layers. The tool facilitates detailed and rapid assessment of API localization within skin strata in under 10 min per skin specimen. To validate this method, three nanoscale dermal drug delivery systems (DDSs) for the antifungal terbinafine that target distinct skin strata—ethosomes, transethosomes, and microemulsion—are designed and characterized. API permeation in human and porcine skin is evaluated using both manual and automated workflows. The integrated approach demonstrates superior accuracy in skin distribution analysis, a substantial reduction in processing time, and improved efficiency in signal‐tissue overlay. Comparative analysis of the DDSs reveals marked differences in drug permeation depth and localization, with transethosomes showing the highest potential for deeper dermal delivery. This method not only provides a powerful tool for DDS evaluation but also enables detailed kinetic studies, offering insights into drug permeation dynamics.

## Graphical abstract

This study presents a novel method for rapidly evaluating dermal drug delivery systems (DDSs). Using mass spectrometry imaging and automated data analysis, it visualizes drug distribution within skin layers in under 10 min. Tested with three nanoscale DDSs for antifungal terbinafine, the approach reveals key differences in drug permeation depth and offers a powerful tool for optimizing dermatological therapies.

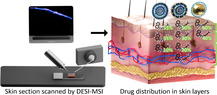

## 1 Introduction

Achieving precise targeting of distinct skin layers (strata) is the holy grail of dermal drug delivery. On the one hand, topical delivery of drugs and cosmeceuticals typically requires crossing the *stratum corneum* (*SC*) for initial permeation, followed by complete retention within the viable epidermis. Conversely, transdermal delivery necessitates ample permeation into the dermis, enabling systemic absorption.^[^ ^1^ ^]^ Yet, even within the dermis, blood vessels density varies between different depths. They are more concentrated in the papillary (upper) dermis compared with the reticular (lower) dermis. The papillary dermis contains a dense network of capillaries that supply nutrients to the epidermis. The reticular dermis has fewer but larger blood vessels.^[^ ^2^ ^]^ Targeting different strata not only aids in achieving local or systemic delivery but also potentially regulates the extent of systemic absorption. Moreover, in topical delivery, the ability to lead an API to the specific skin depth and accumulate it there can greatly improve the efficacy of the treatment.^[^ ^3^ ^]^

Fungal infection is a good example of a condition, in which the depth of API permeation into the skin is essential. Fungi usually invade the skin from the surface leading to “superficial” mycoses and gradually progress into deeper skin layers causing “cutaneous” and “subcutaneous” mycosis. Each fungal species eventually homes at a particular depth. For example, species of *Candida albicans* and *Pitryiasis versicolor* infections cause the most superficial infection affecting mostly the epidermis, while species in *Sporotrichosis*, *Mycetoma*, and *Chromoblastomycosis* affect the dermis and often spread to the hypodermis.^[^ ^4^ ^]^ Since different fungi species reside in different skin strata, there is a need to deliver and localize antifungals to varying depths with high precision.

The main obstacle to developing delivery systems that target specific skin layers is the lack of direct assessment of permeation depth. Currently, the conventional workflow of *in vitro* permeation testing studies for dermal drug formulations includes detecting the amount of drug permeated through *ex vivo* skin mounted on Franz diffusion cell using liquid chromatography.^[^ ^5^ ^]^ However, this method primarily provides information on drug flux across the skin. While it can be extended to assess drug accumulation in different skin strata by extracting the drug from each layer separately, this approach is less direct, more labor‐intensive, and potentially susceptible to human error due to multiple handling steps. Alternatively, in the case of fluorescent APIs, confocal laser scanning microscopy (CLSM) can be employed to detect and quantify their localization at various skin depths.^[^ ^6^ ^]^ Due to the relative rarity of fluorescent APIs, this evaluation method heavily relies on the use of fluorescent model molecules. These model molecules can be incorporated into the evaluated DDS either in lieu or alongside the API, and their distribution in various skin depths is eventually measured by the CLSM.^[^ ^7^ ^]^ Although these fluorescent molecules are typically selected to have similar physicochemical characteristics to the API, the results directly pertain to the distribution of the model molecule and not of the actual API. Alternatives to this analysis include measuring the skin distribution of fluorescently labeled API.^[^ ^6^ ^]^ However, fluorescent labeling often markedly increases the size of the molecule and changes its basic physicochemical properties, hindering the reliability of distribution results.

To overcome these challenges, implementing more direct methods for spatially visualizing drugs in the skin, such as confocal Raman spectroscopy (CRS) and mass spectrometry imaging (MSI)‐based techniques, can be considered. While CRS is a noninvasive, *in vivo* tool with high spatial resolution and minimal sample preparation, it has limitations in sensitivity, broad chemical detection, and molecular identification.^[^ ^8^, ^9^ ^]^ In contrast, MSI techniques offer direct detection of drugs (after ionization) within tissue sections, enabling precise visualization of drug distribution across skin layers. The MSI techniques employed to date to detect API distribution in the skin include matrix‐assisted laser desorption ionization MSI (MALDI‐MSI),^[^ ^10^ ^]^ time‐of‐flight secondary ion mass spectrometry (TOF‐SIMS),^[^ ^11^ ^]^ and desorption electrospray ionization MSI (DESI‐MSI).^[^ ^12^, ^13^, ^14^, ^15^ ^]^ These techniques differ in the range of detectable molecules, the extensiveness of sample preparation, spatial resolution, tissue scanning time (duration of the measurement), and ambient conditions.^[^ ^16^ ^]^ To leverage the advantages of both approaches, Belsey et al. combined Raman spectroscopy with SIMS, enhancing precision and spatial resolution while adding complexity to the study of skin permeation.^[^ ^17^ ^]^ Among MSI techniques, DESI‐MSI, in particular, allows relatively rapid imaging of unprocessed skin sections and direct visualization of a wide variety of molecules in the skin. With the recent advances in spatial resolution of DESI‐MSI, this method can reliably resolve 5–50 μm rendering its suitability for delineation of skin layers.^[^ ^18^, ^19^, ^20^, ^21^ ^]^ Despite the potential of MSI techniques to aid in the development of dermal DDSs, the research on skin permeation studies using those techniques remains limited, with approximately a dozen reports available.^[^ ^12^, ^13^, ^17^, ^22^, ^23^, ^24^, ^25^, ^26^, ^27^, ^28^, ^29^, ^30^, ^31^ ^]^ All these studies lack a systematic approach to provide quantitative feedback on drug localization across different skin layers, hindering their implementation in dermal DDS evaluation. One of the primary challenges is the time‐consuming and complex analysis of imaging data. Precisely overlaying the mass spectrometry signal of the drug with other spectral and anatomical features of each skin layer and sublayer requires significant expertise and can take anywhere from several hours to a few days per skin specimen, depending on factors such as section size, imaged region, and spatial resolution.

**Figure 1:**
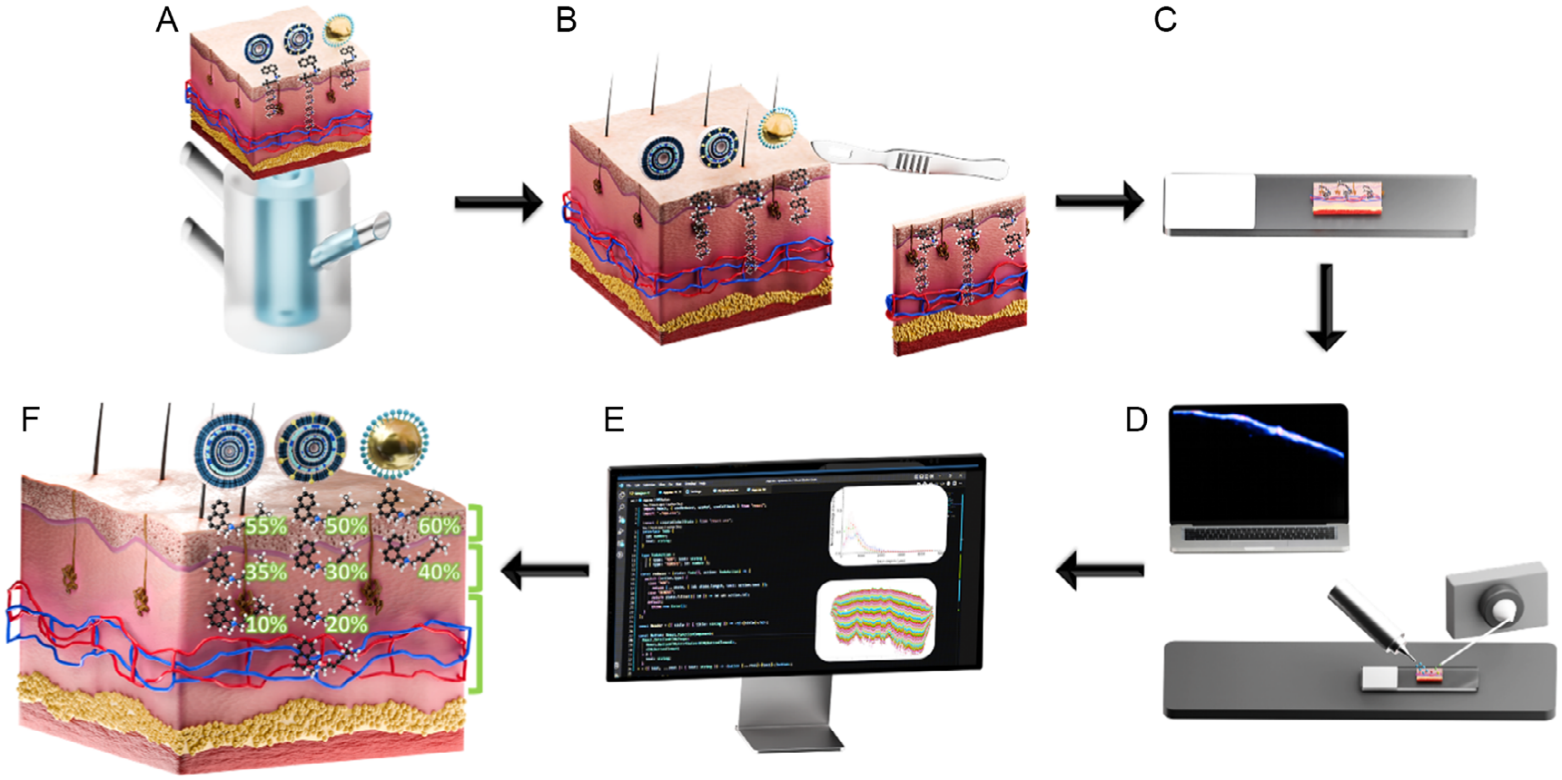
Rapid and direct method for visualizing and comparatively quantifying API distribution across different skin depths. A) Excised skin square is mounted on Franz diffusion cell for DDS application. B). Treated skin square is sectioned vertically in a cryostat. C) Skin section is mounted on the glass slide. D) Skin section is scanned with DESI‐MSI. E) Data are processed manually or automatically. F) API distribution across skin layers visualized and quantified.

In this study, we developed a rapid and direct method for visualizing and comparatively quantifying API distribution across different skin depths based on DESI‐MSI (**Figure** **1**). We introduced a novel automatic tool based on spatial clustering designed to overcome overlaying challenges, reduce analysis time, and improve accuracy, thereby facilitating quantitative feedback. This tool separates skin tissue‐related MSI signals from the signals of the background and automatically divides skin MSI data into thin layers of predefined width starting from the *SC*. This process recapitulates skin strata with high precision and enables a full evaluation of drug distribution in skin layers within a maximum processing time of 10 min per specimen. Since the tool is based on processing raw mass spectrometry data, it is expected to be compatible with a variety of MSI techniques, as above. To ensure reproducibility and reliability, standardization of this method was performed and its performance was compared against manual data processing. At first, nanoscale delivery systems of antifungal terbinafine (TBF) were designed to target different skin strata. Then, their performance was evaluated in skin from different sources (human and porcine) employing both automatic and manual processing methods and shedding light on skin permeation depth and localization within various skin strata. Conventional CLSM analysis was also carried out. Finally, leveraging the efficiency of the automated processing method, the permeation kinetics was evaluated. Thus, this approach not only expedites data analysis but also allows monitoring of skin delivery dynamics leading to a better understanding of the permeation process and providing immediate feedback on delivery system effectiveness in targeting specific skin layers. This has important implications for both safety and efficacy, e.g., improving the precision of antifungal treatments and reducing systemic exposure to potentially harmful compounds, such as corticosteroids and retinoids. To the best of our knowledge, such a direct, label‐free, and rapid method for assessing topical drug distribution to optimize delivery strategies is not currently achievable using established techniques.

## 2 Results and Discussion

### 2.1 Preparation and Characterization of DDSs

In this study, we developed three nanoscale DDSs for antifungal drug delivery: ethosomes, transethosomes, and a microemulsion. While all these systems have the potential to permeate their API cargo into the skin, they are expected to differ in their ability to reach various skin strata.^[^ ^32^, ^33^, ^34^, ^35^ ^]^ Ethosomes are multilamellar soft vesicles composed of phospholipids, water, and high concentration of ethanol, forming bilayers throughout their structure. Ethanol interacts with membrane lipids, increasing fluidity and facilitating ethosomal permeation across the *SC* into the deeper epidermal layers.^[^ ^36^, ^37^, ^38^ ^]^ Ethosomes have been reported to reach the viable epidermis.^[^ ^39^ ^]^ Transethosomes are modified ethosomes that include an edge activator, a lower ethanol concentration, and an additional permeation enhancer.^[^ ^40^ ^]^ These ultradeformable vesicles show excellent potential for deeper skin drug delivery and improved stability. They have been reported to permeate deeper than ethosomes,^[^ ^41^ ^]^ although the exact depth of their permeation is not well established.^[^ ^33^ ^]^ Microemulsions are thermodynamically stable, nanometric dispersive systems of oil, surfactants, and water, capable of incorporating APIs. Microemulsions have been reported to primarily deliver APIs to the *SC*,^[^ ^42^ ^]^ although experiments using Franz cells often show the presence of the drug in the receiver phase, suggesting permeation across the skin.^[^ ^34^ ^]^ For this study, TBF was selected as the model antifungal drug. TBF is a poorly water‐soluble compound with a LogP of 5.6 and a broad‐spectrum antimycotic activity. All DDSs were prepared with 1% w/w of TBF.

**Figure 2:**
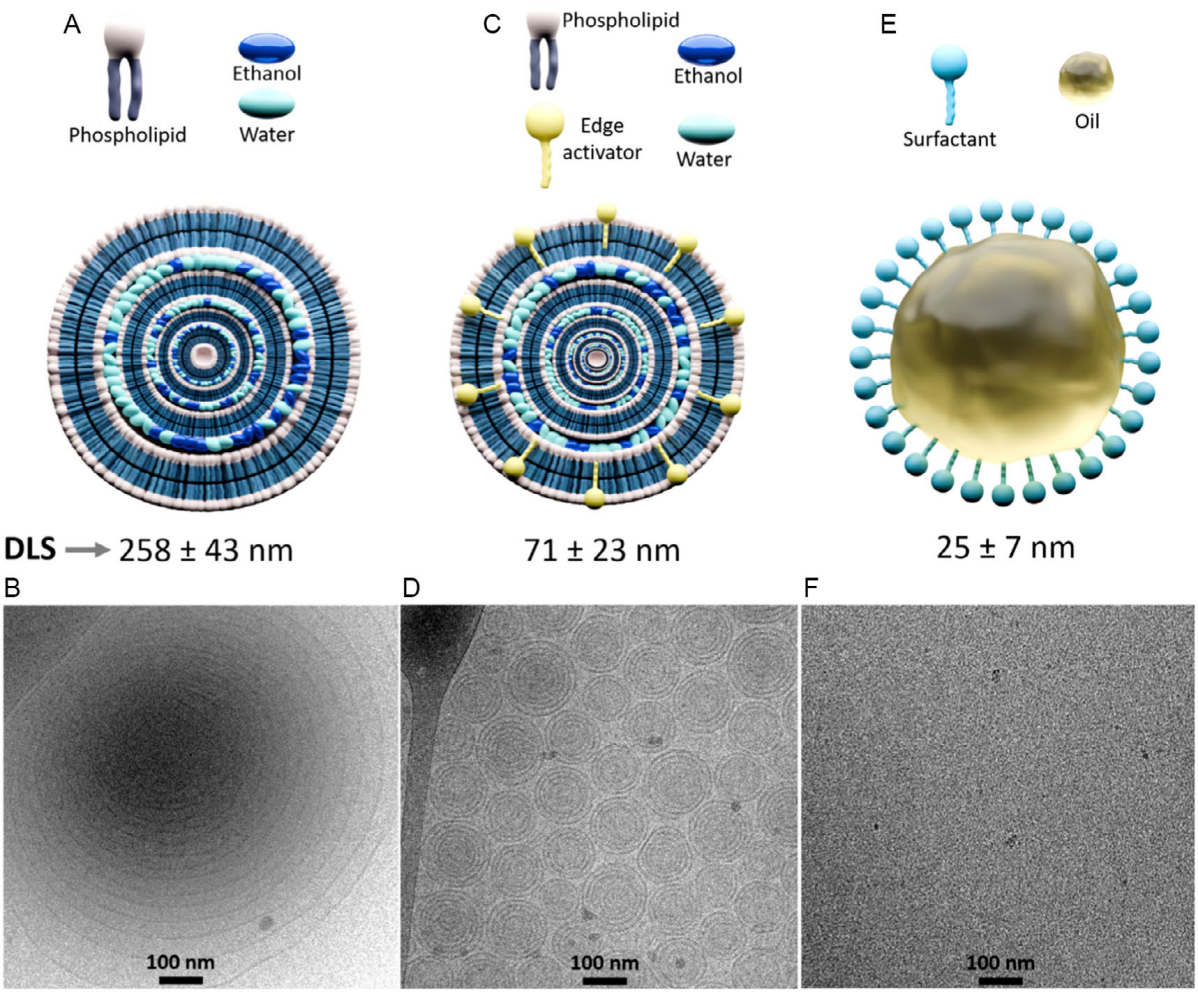
Illustrations and characterization of the three DDSs containing TBF: A) Schematic illustration of the ethosomal DDS. B) Cryo‐TEM image of the ethosomal DDS. C) Schematic illustration of the transethosomal DDS. D) Cryo‐TEM image of the transethosomal DDS. E) Schematic illustration of the microemulsion DDS. F) Cryo‐TEM image of the microemulsion DDS. The average particle size of each DDS, measured by DLS, is displayed below the corresponding illustration. Additional DLS data and cryo‐TEM images can be found in S1, and S2 respectively, Supporting Information.

The ethosomal system for TBF delivery was designed with 50% w/w ethanol to enhance skin permeation. Propylene glycol (20% w/w) was included as a secondary permeation enhancer, and vitamin E acetate (0.2% w/w) was added as an antioxidant. The average vesicle size, measured by dynamic light scattering (DLS), was 258 ± 43 nm (**Figure** **2**A). Cryo‐transmission electron microscope (cryo‐TEM) analysis (Figure 2B) revealed the multilamellar nature of the vesicles. These multilamellar structures can rearrange themselves depending on dilution prior to measurement and the temperature, which differ in cryo‐TEM and DLS measurements. The DLS is considered to provide a more accurate size, while cryo‐TEM enables visual assessment of the lamellar structures. Additionally, the cryo‐TEM measurements were conducted a few weeks after the DLS measurements. Over time, the dynamics of the multilamellar structures can cause an increase in both their size and polydispersity.^[^ ^41^, ^43^, ^44^, ^45^ ^]^

We further developed a transethosomal DDS for TBF, incorporating cinnamaldehyde (0.3% w/w) as an edge activator, Tween 80 (5% w/w), ethanol (17.9% w/w), and propylene glycol (10.1% w/w) as permeation enhancers. Compared to ethosomes, this formulation contains lower ethanol and propylene glycol levels, compensated by the inclusion of the edge activator and an additional permeation enhancer. Furthermore, it has a higher water‐to‐ethanol ratio. The average vesicle size, measured by DLS, was 71 ± 23 nm (Figure 2C). Cryo‐TEM analysis (Figure 2D) shows multilamellar vesicles (about 100 nm in diameter). These are smaller compared to ethosomes and have consistent spacing between the lamellar layers, which creates more ordered structures.

Finally, a TBF‐loaded microemulsion was formulated using 5.8% w/w oleic acid, 26.6% w/w Labrasol, 26.6% w/w Transcutol P as the inner oil phase and amphiphiles, and 40% w/w water. Oleic acid, a natural skin component, acted as a permeation enhancer. To retain the drug within the skin, the water content was kept below 55%. The droplet size of the microemulsion, determined by DLS, was 25 ± 7 nm (Figure 2E). Cryo‐TEM characterization (Figure 2F) revealed the characteristic pattern typical of microemulsions,^[^ ^46^ ^]^ with small droplets of about 5–7 nm.

Eventually, we compared the ability of the DDSs described above to permeate TBF into the skin by evaluating TBF signal intensity at four different skin depths, which approximately correspond to anatomical abdominal skin strata:^[^ ^47^, ^48^ ^]^ 100 μm—corresponding to the viable epidermis, 500 μm—corresponding to the upper papillary dermis, 1500 μm—corresponding to the lower reticular dermis, and 1500 μm and above—corresponding to the hypodermis. When comparing permeation into porcine ear skin that comprises thinner dermis stratum, some of these comparison points were omitted.

### 2.2 Evaluation of DDSs Using CLSM

**Figure 3:**
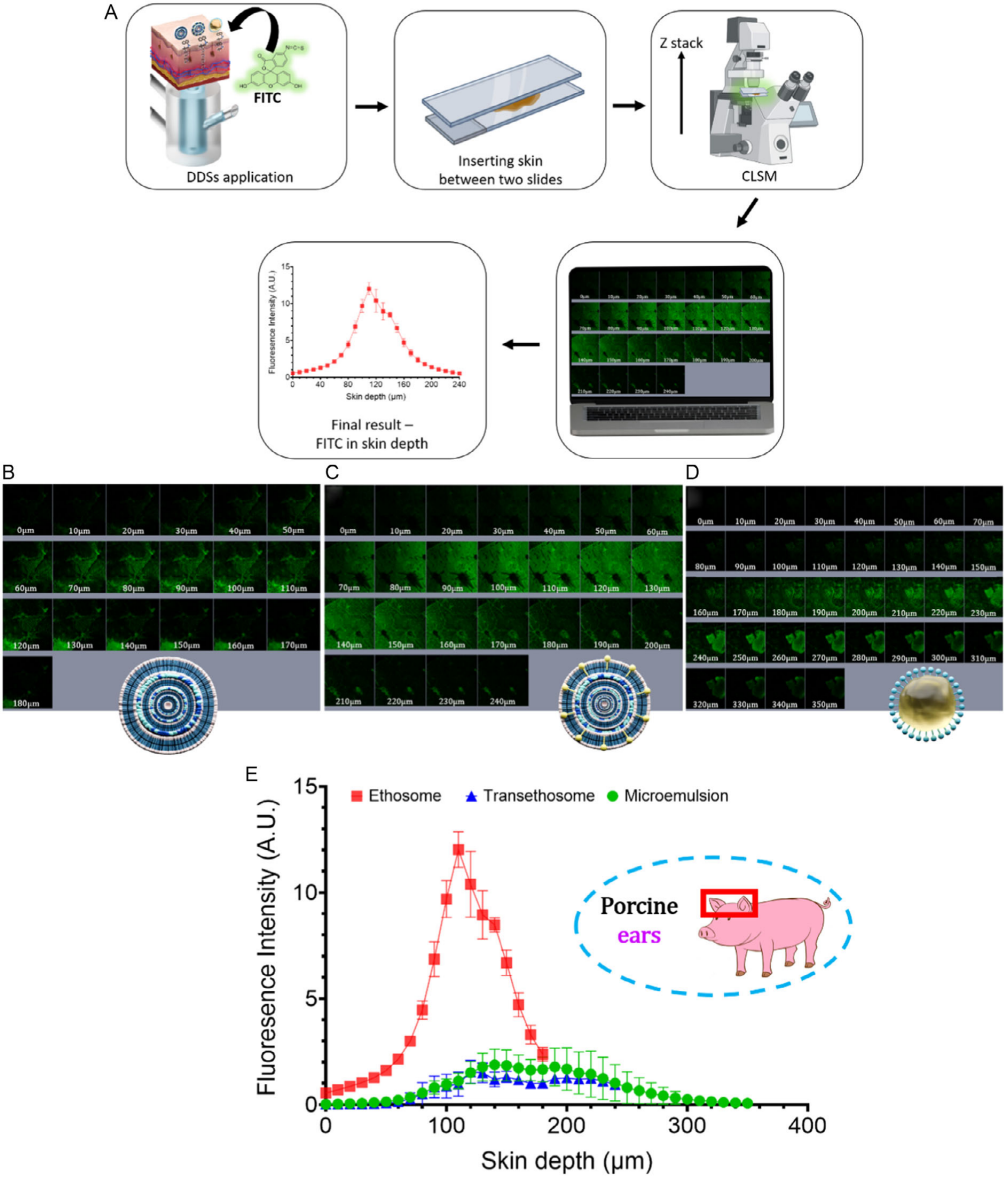
A) Experimental workflow: DDSs containing both FITC and TBF were applied to porcine ear skin squares equilibrated on Franz cells. The treated skin squares were mounted between two glass slides, and fluorescence signals were recorded in Z‐stack mode using CLSM. Each layer along the *Z*‐axis is represented by an image, and fluorescence intensity is plotted against skin depth. B–D) Fluorescence images in Z‐stack mode for (B) ethosomal DDS, (C) transethosomal DDS, and (D) microemulsion DDS. E) Comparison of fluorescence intensity signals of FITC delivered by the three DDSs versus skin depth.

To assess the ability of the designed DDSs to deliver their cargo into the skin, we conducted conventional CLSM analysis using fluorescein isothiocyanate (FITC, LogP = 5.3) as a fluorescent model molecule^[^ ^49^, ^50^, ^51^, ^52^, ^53^ ^]^ (**Figure** **3**A). FITC was incorporated into each DDS at a concentration of 1% w/w, matching the TBF concentration. Porcine ear skin squares were equilibrated on Franz cells and subsequently analyzed with CLSM to evaluate fluorescence at varying skin depths. The zero and endpoint fluorescence signals were determined relative to the *SC*, assuming substantial signal presence at the *SC* layer. Figure 3B–D presents fluorescence images for each DDS, recorded using CLSM in z‐stack mode. The fluorescence intensity signals were quantified and converted into arbitrary units (AUs) using ImageJ software, and the results are compared in Figure 3E.

The results indicate that the ethosomal DDS exhibited the best performance, with the strongest fluorescence signal detected up to a depth of 200 μm. Comparatively, the microemulsion permeated FITC slightly deeper than the transethosomal DDS, as shown in Figure 3E. Ethosomes have been reported to enhance delivery of compounds to the skin layers due to their exceptional capability to fuse with the *SC* lipids.^[^ ^45^ ^]^

### 2.3 Establishing TBF Detection and Manual Calculation in Skin Layers Using DESI‐MSI

After evaluating the skin fluorescence signal using CLSM, we sectioned the skin squares vertically into 20 μm thick sections to reveal the skin strata. These sections were mounted on microscope slides and analyzed using DESI‐MSI. **Figure** **4**A illustrates the workflow for utilizing DESI‐MS images of skin sections to perform manual calculations. In this study, a spatial resolution of 50 μm was used to establish the workflow, though higher resolutions could be employed in the future, leveraging the shorter processing time enabled by the new tool. While imaging at a 50 μm spatial resolution has limited ability to correlate the distribution of TBF with intricate skin structures (e.g., anatomical sublayers of the epidermis), fungal infections can spread across the entire skin. Therefore, in these experiments, the full depth of skin specimens was imaged, prioritizing overall skin coverage over fine morphological details. Notably, DESI‐MSI is capable of imaging at a spatial resolution of up to 5 μm (pixel size), allowing for the effective delineation of 5 μm layers. We demonstrate it in high‐resolution imaging (5 μm pixel size) of TBF permeation by transethosomal DDS into human abdominal skin allowing delineation of drug intensities in *SC* and epidermis sublayers (Figure S3, Supporting Information).

Figure 4A) Experimental workflow for the manual calculation of TBF in porcine ear skin layers using HDI software. This includes image overlay and ROI annotations for each layer. B) Process description for the ethosomal system. C) DESI‐MSI distribution image of TBF (*m/z* 292.207, [M + H]^+^) with a pixel resolution of 50 μm. D) Histological image of a vertically sectioned, H&E‐stained porcine ear skin sample, with the *SC* located at the bottom. E) Overlay of the DESI‐MSI distribution image (C) and the histological image (D) with 60% transparency applied to the DESI‐MSI image, created using HDI software. F–N) Nine consecutive manually defined ROI layers, each with a thickness of one pixel (50 μm). The *SC* direction is indicated in the images. A graph represents the average normalized intensity of TBF in each ROI layer after normalization by sum (as detailed in S7, Supporting Information), with colors corresponding to the respective ROI layers. The same methodology was applied to the other DDSs. O) Results for the transethosomal system. P) Overlay of DESI‐MSI and histological images for the transethosomal system. Q) ROI annotations created for the transethosomal system. Scale bar: 1 mm. R) Results for the microemulsion system. S) Overlay of DESI‐MSI and histological images for the microemulsion system. T) ROI annotations created for the microemulsion system. Scale bar: 1 mm. U) Comparative graph summarizing TBF permeation into porcine ear skin for the three DDSs. Skin strata are indicated as: epidermis (*), upper dermis (**), and lower dermis (***). The hypodermis was not present in the porcine ear skin samples.

**Figure:**
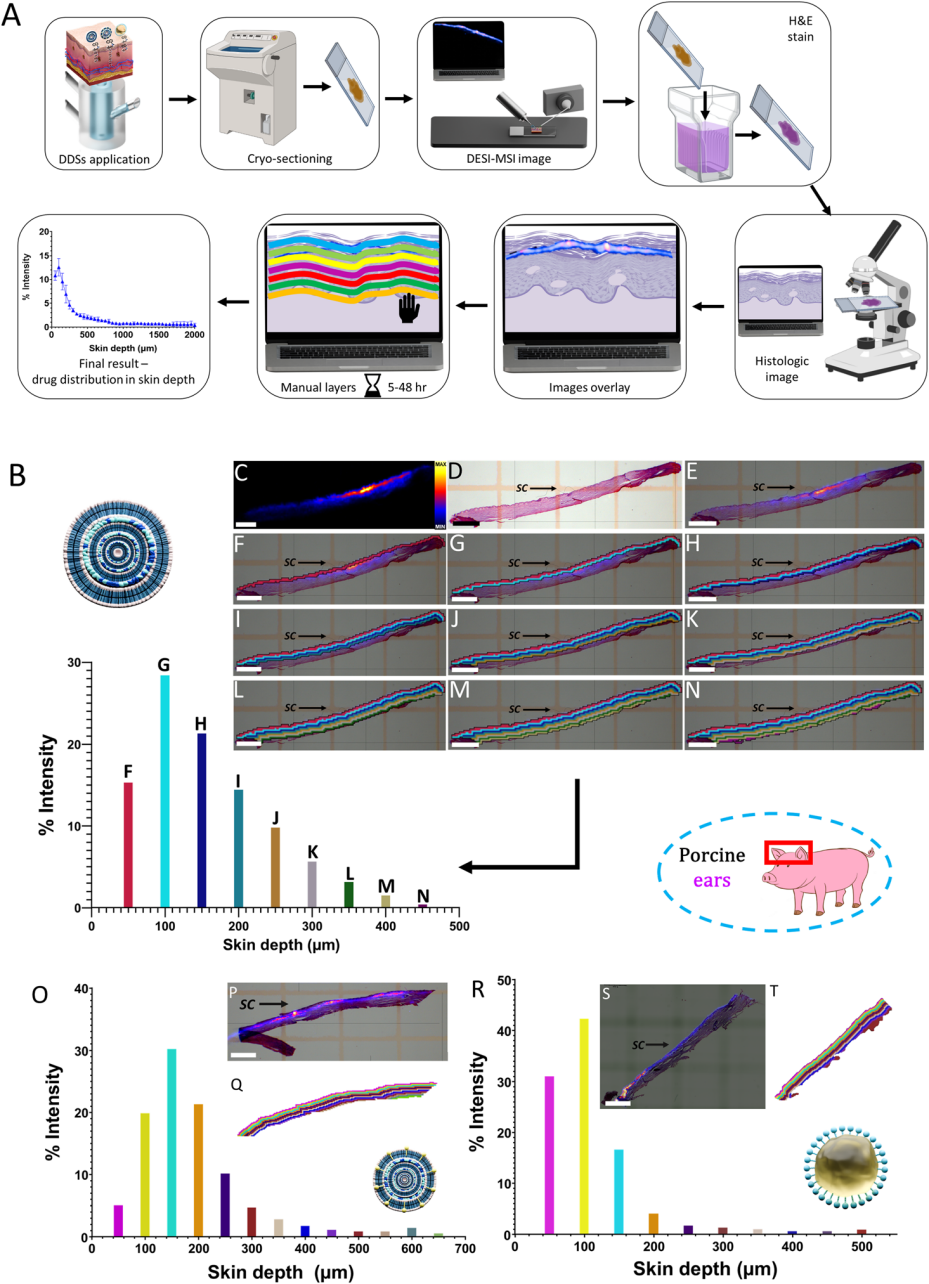

**Figure:**
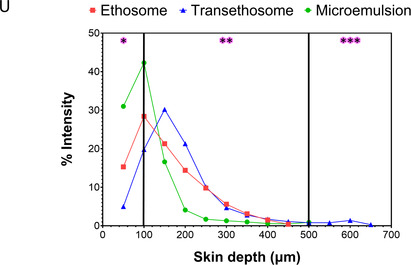

The TBF signal was detected as a protonated adduct at *m/z* 292.207 in a positive ionization mode (Figure S4, Supporting Information), and MS images showing its distribution in the skin sections were generated (Figure 4). These images, displayed as heat maps, use yellow to indicate the highest TBF signal intensity, blue for the lowest, and black to represent signal absence. To confirm that signals at the target *m/z* are specific to TBF and are not due to background or endogenous skin components, a control experiment was performed using drug‐free transethosomal DDS. As shown in the spectrum (Figure S5, Supporting Information), no peaks were observed at the relevant *m/z*, indicating the absence of interfering signals. For instance, the ethosomal DDS (Figure 4B) produced the MS image presented in Figure 4C. Following DESI‐MSI, the same skin sections were stained using a modified hematoxylin and eosin (H&E) staining protocol for unfixed tissues (S6, Supporting Information) to obtain histological images of the skin (Figure 4D). These histological images were then overlaid with DESI‐MSI drug distribution images to precisely locate the drug within the skin structure (Figure 4E). To further analyze the data, the images were divided into layers of controlled width using the ROI (region of interest) selection tool in the High‐Definition Imaging (HDI) software. The ROI width was set at 50 μm, and pixel‐by‐pixel marking was employed to accurately delineate the *SC* and the deeper skin layers. The first ROI layer (Figure 4F) contained the *SC*, which in porcine ear skin is ≈7 μm thick, with the remaining portion consisting of the viable epidermis, ≈45 μm thick.^[^ ^31^ ^]^ Consequently, the first two ROI layers (Figure 4G), representing 100 μm of skin, encompassed both the *SC* and the viable epidermis. The detection of TBF signal in the third ROI layer and beyond (Figure 4H–N) indicated that the drug had permeated into the dermis. Due to the relative thinness of porcine ear skin, fewer ROI layers (maximum of 13 per skin section) were required compared to skin from other sources. This characteristic reduced the processing time, making porcine ear skin an optimal choice for these experiments. A summary graph demonstrates the average signal intensities across all ROI layers, representing TBF skin permeation by the ethosomal DDS. Using DESI‐MSI for calculation, we observed that the ethosomal DDS facilitated TBF permeation as deep as 450 μm into porcine ear skin, whereas CLSM detected FITC permeation up to a depth of 240 μm. This finding highlights the advantage of direct molecular distribution evaluation through MSI.

### 2.4 Comparative Analysis of Different DDSs Using Manual Calculation

After establishing the calculation method by manually selecting ROIs for the ethosomal DDS, we extended the evaluation to include TBF permeation from two additional DDSs: the transethosomal DDS (Figure 4O, with overlay and ROI images shown in Figure 4P,Q, respectively) and the microemulsion DDS (Figure 4R, with overlay and ROI images in Figure 4S,T, respectively). Figure 4U provides a comparative analysis of TBF permeation across the three systems. The microemulsion DDS permeated TBF to a depth of 200 μm, with the highest drug signal detected between 50 and 100 μm. This indicates that the microemulsion primarily facilitates drug accumulation in the epidermis. In contrast, the ethosomal and transethosomal DDSs achieved TBF permeation to a depth of 400 μm, successfully reaching the upper dermis. Notably, the ethosomal formulation exhibited the highest drug signal at a depth of 100–150 μm, whereas the transethosomal formulation showed peak drug signal at 150–200 μm. These results suggest that: (1) the transethosomal DDS achieves the greatest skin depth of TBF permeation, displaying a distinct distribution pattern compared to FITC, and (2) this method provides direct, quantitative insights into drug distribution patterns, demonstrating its potential for designing DDSs tailored to target specific skin layers.

### 2.5 TBF Permeation Profiles in Skin from Different Sources

**Figure 5:**
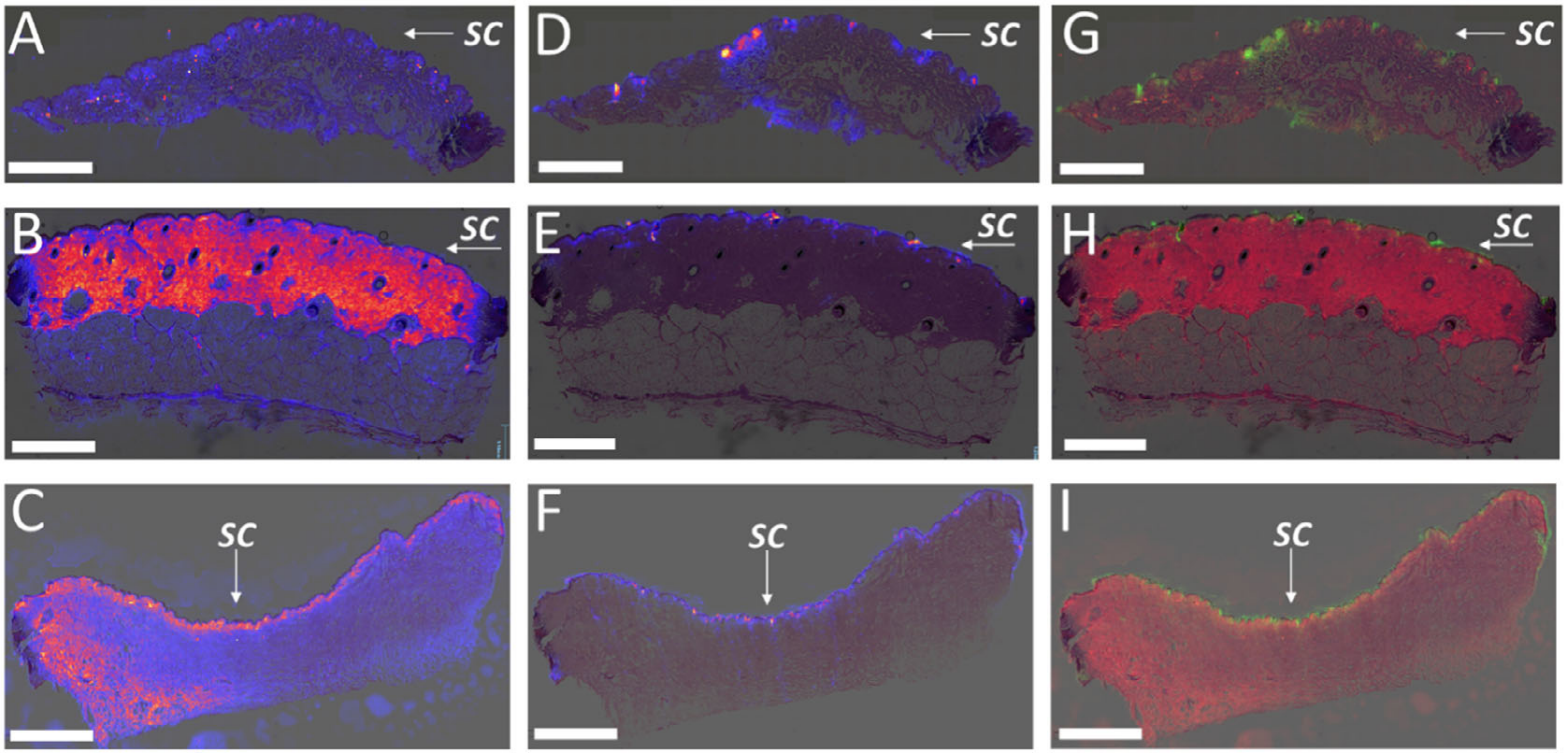
Identification of the choline peak to improve overlay accuracy between histological images and DESI‐MSI TBF distribution images for the manual ROI layers method. A–C) Distribution images of choline (*m/z* 104.107, [M]^+^) overlaid with histological images of skin: (A) porcine ear skin, (B) porcine abdominal skin, and (C) human abdominal skin. D–F) Distribution images of TBF (*m/z* 292.207, [M + H]^+^) overlaid with histological images of skin: (D) porcine ear skin, (E) porcine abdominal skin, and (F) human abdominal skin. G–I) Combined overlay of TBF (*m/z* 292.207, green) and choline (*m/z* 104.107, red) distribution images with histological images of skin: (G) porcine ear skin, (H) porcine abdominal skin, and (I) human abdominal skin. The scale bar for porcine ear and porcine abdominal skin images is 2 mm, while the scale bar for human abdominal skin images is 3 mm. All images shown are of the ethosomal DDS series as an example. The *SC* direction is indicated in all images.

Next, we compared the permeation profiles of TBF across three types of skin: porcine ear skin, porcine abdominal skin, and human abdominal skin. To reliably conduct permeation experiments in thicker skin sections using MSI, we needed to enhance the precision of superimposing MS images with corresponding histological images. Figure 4U demonstrates that TBF does not permeate throughout the entire thickness of porcine ear skin sections, leaving drug‐free ROIs. This creates challenges in overlaying MS and histological images, particularly when dealing with thicker skin, such as abdominal skin. To overcome this, we identified a consistent peak present across all skin layers in positive ion mode. This peak, detected at *m/z* 104.107, was identified as a positive ion of the endogenous molecule choline based on isotopic pattern analysis and tandem MS (Figure S8, Supporting Information). Choline, as an organic cation, is required by epidermal keratinocytes for optimal proliferation and differentiation.^[^ ^54^, ^55^ ^]^ Choline serves as substrate for the synthesis of several skin metabolites (e.g., phosphatidylcholines, sphingomyelin, and glycerophosphorylcholines), and was detected in all three skin types: porcine ear skin (**Figure** **5**A), porcine abdominal skin (Figure 5B), and human abdominal skin (Figure 5C). The choline distribution image served as a reliable marker for precise alignment of MS images with skin histological sections. Using this approach, we confirmed the exact location of TBF distribution in skin sections (Figure 5D–F). The final overlay images, integrating TBF distribution with histological data, are presented in Figure 5G–I.

**Figure 6:**
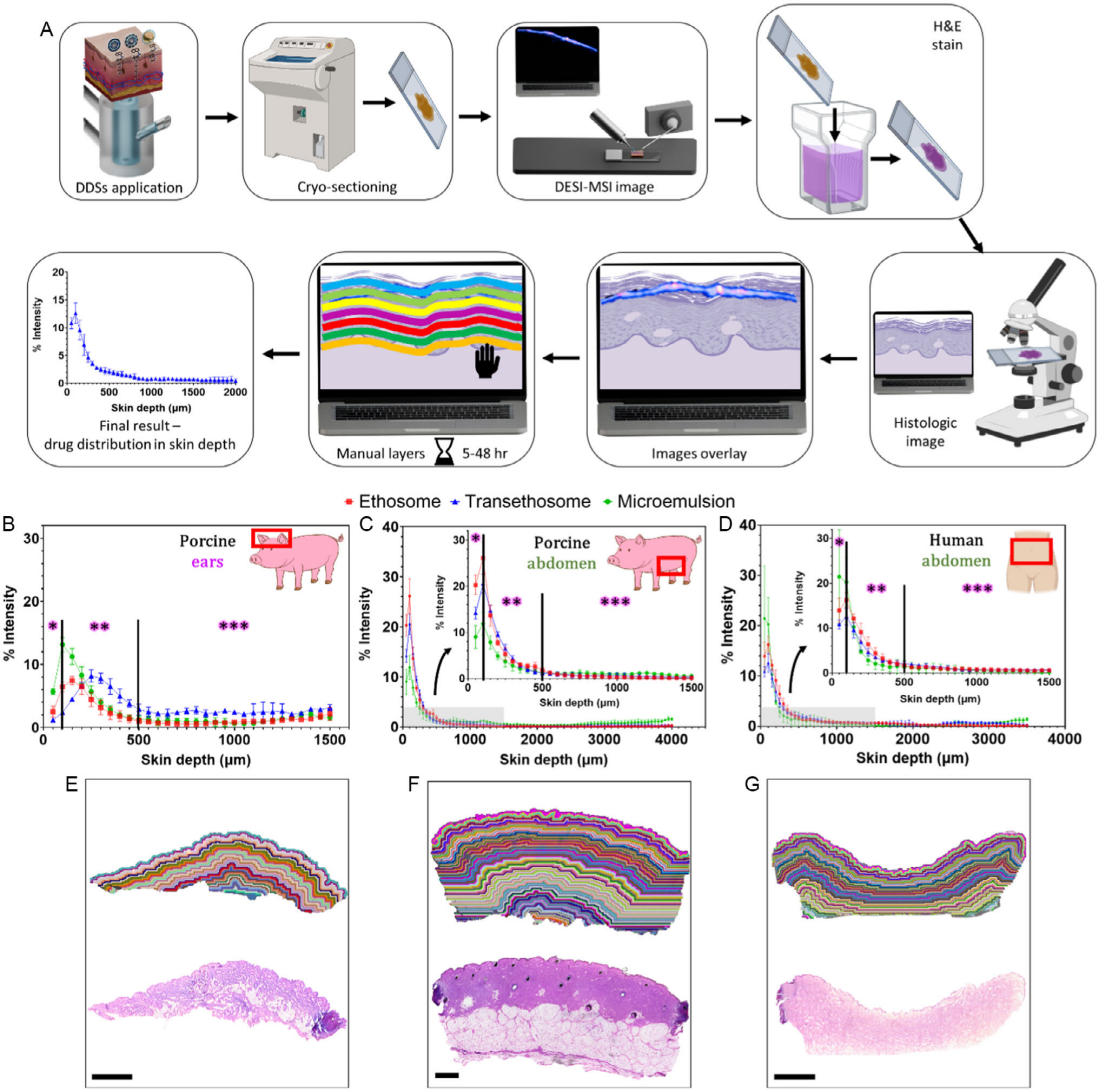
Comparison of TBF skin permeation across three different DDSs and three different skin sources, calculated manually using HDI software. A) Flow chart of the experimental process. B–D) Graphical representation of TBF permeation to skin, using three DDSs: (B) porcine ear skin, (C) porcine abdominal skin, and (D) human abdominal skin. E–G) Skin sections manually divided into artificial layers using the ROI tool in HDI software, with each layer having a thickness of 50 μm: (E) porcine ear skin (scale bar: 2 mm), (F) porcine abdominal skin (scale bar: 1 mm), and (G) human abdominal skin (scale bar: 3 mm). Histological images of the skin sections are shown beneath each corresponding ROI image. All images shown are of the ethosomal DDS series as an example (additional details in S9, Supporting Information). Tissue layer images are oriented with the *SC* at the top; *epidermis, **upper dermis, and ***lower dermis.

Next, we evaluated the TBF permeation profiles facilitated by the DDSs using the manual method across skin from different sources (**Figure** **6**A). Figure 6B presents TBF permeation into porcine ear skin from the three DDSs, showing that the transethosomal DDS achieved the greatest skin penetration depth. However, the permeation patterns differed among the three DDSs when tested on porcine and human abdominal skin (Figure 6C,D, respectively). In porcine ear skin, TBF delivered by the transethosomal DDS permeated to a depth of 500 μm. In contrast, in porcine and human abdominal skin, TBF permeation by all three DDSs was restricted to the first 300 μm. These differences may be attributed to variations in skin thickness. Thinner skin, such as porcine ear skin, equilibrates more quickly in Franz cells, bringing the upper skin layers into closer proximity to the receiver solution, which can influence the permeation rate. Thicker skin, on the other hand, provides a more realistic model for evaluating drug delivery into the skin. However, processing thicker skin is significantly more labor‐intensive due to the increased number of ROI layers required for analysis. In this study, porcine ear skin sections contained ≈40 ROI layers (Figure 6E), whereas porcine and human abdominal skin sections contained around 80 ROI layers each (Figure 6F,G, respectively), necessitating manual pixel‐by‐pixel segmentation.

### 2.6 TBF Calculation Using an Automated Method

While the manual ROI method provides valuable insights, it has several limitations. First, it relies on manual pixel selection, which is prone to human error. Second, it is both time‐ and labor‐intensive, especially when analyzing thicker or irregular skin sections. Third, the method depends on accurate superimposition of MS and histological images, often requiring identification of endogenous ions, such as choline (as described above). To address these challenges, we developed an automated approach for segmenting skin into artificial, size‐controlled layers using only MS data. The algorithm and its underlying methodology are described below.

We have designed a web‐based Python platform for processing MSI data. For access to the algorithm's code (available in a Google Notebook) and the user manual, please refer to S10, Supporting Information. The platform requires minimal user input, including data quality assessment and the presetting of certain parameters. MSI data are uploaded to the platform in .txt format, which can be stored on Google Drive for user convenience.

**Figure 7:**
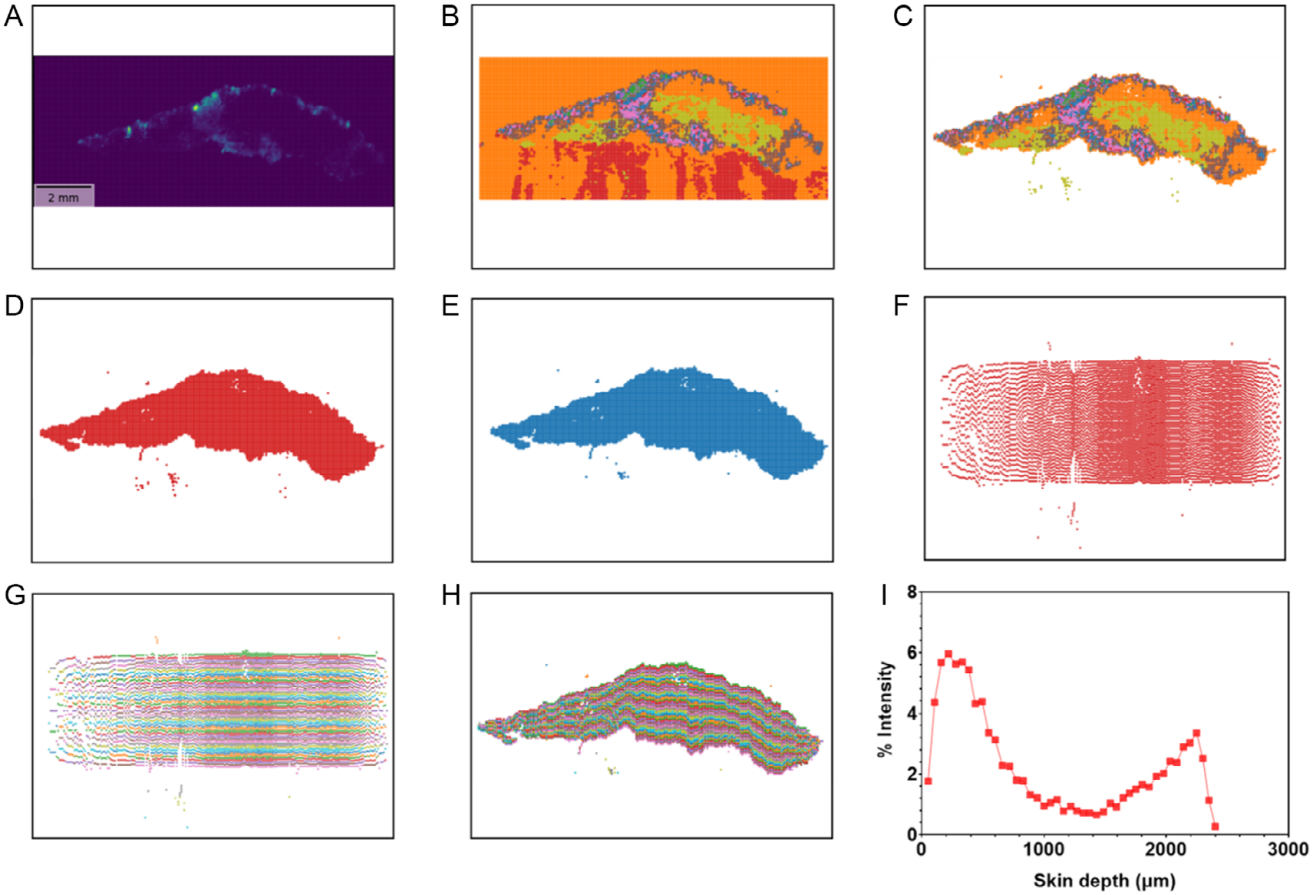
Automated method for calculation of TBF permeation facilitated by the ethosomal DDS in porcine ear skin layers. A) *m/z* 292.207 distribution image (for data quality evaluation). Scale bar: 2 mm. B) KMeans clustering based on MS data, with *k* = 15. C) Selected clusters representing tissue. D) Full tissue visualization after cluster selection. E) Final tissue image after cropping outliers. F) Straightened tissue prepared for division into XY layers. G) Tissue divided into 50 XY layers, each ≈1 pixel thick (50 μm). H) Reprojection of each layer onto the original tissue shape. I) Final graph showing the average intensity of TBF (*m/z* 292.207) in each XY layer, normalized by sum. All images are oriented with the *SC* at the top.

The workflow begins by verifying the presence of the MS image of the peak of interest in the dataset and ensuring the data is not corrupt. Visualization of the data should display a pattern similar to that shown in **Figure** **7**A. The MSI data signal is then used to divide the image into preset number of clusters based on chemical content (Figure 7B). Details of this optimization process are provided in S11, Supporting Information. With user input, the skin tissue is delineated from the background by identifying and excluding irrelevant clusters (Figure 7C). The final tissue outline is generated (Figure 7D), and outliers are removed (Figure 7E).

To divide the skin into controlled artificial depth layers, the tissue is “straightened” and “widened” through a linear manipulation of the pixel *y*‐axis values: (Figure 7F): *Y* = (*Y* – Avg(*Y*))/(Var(*Y*)) and then *Y* = *Y* * 100 000. This new shape allows for the automatic division of the tissue into a user‐defined number of depth‐equivalent layers (Figure 7G). Each layer is mapped back to the original tissue shape (Figure 7H). The average width of each layer is determined by the average number of pixels across the layer (with 1 pixel corresponding to 50 μm, in our data). The average signal of the drug in each layer is normalized by dividing the intensity of drug's *m/z* peak in a specific layer by the sum of the drug's *m/z* peak intensities across all layers (normalization by sum, see S12, Supporting Information). This normalization generates a plot of drug permeation into the skin, assessing its distribution at different skin depths (Figure 7I). The entire data processing workflow takes ≈10 min to complete.

### 2.7 Comparison of TBF Permeation into Skin Using Different DDSs and the Automated Method

**Figure 8:**
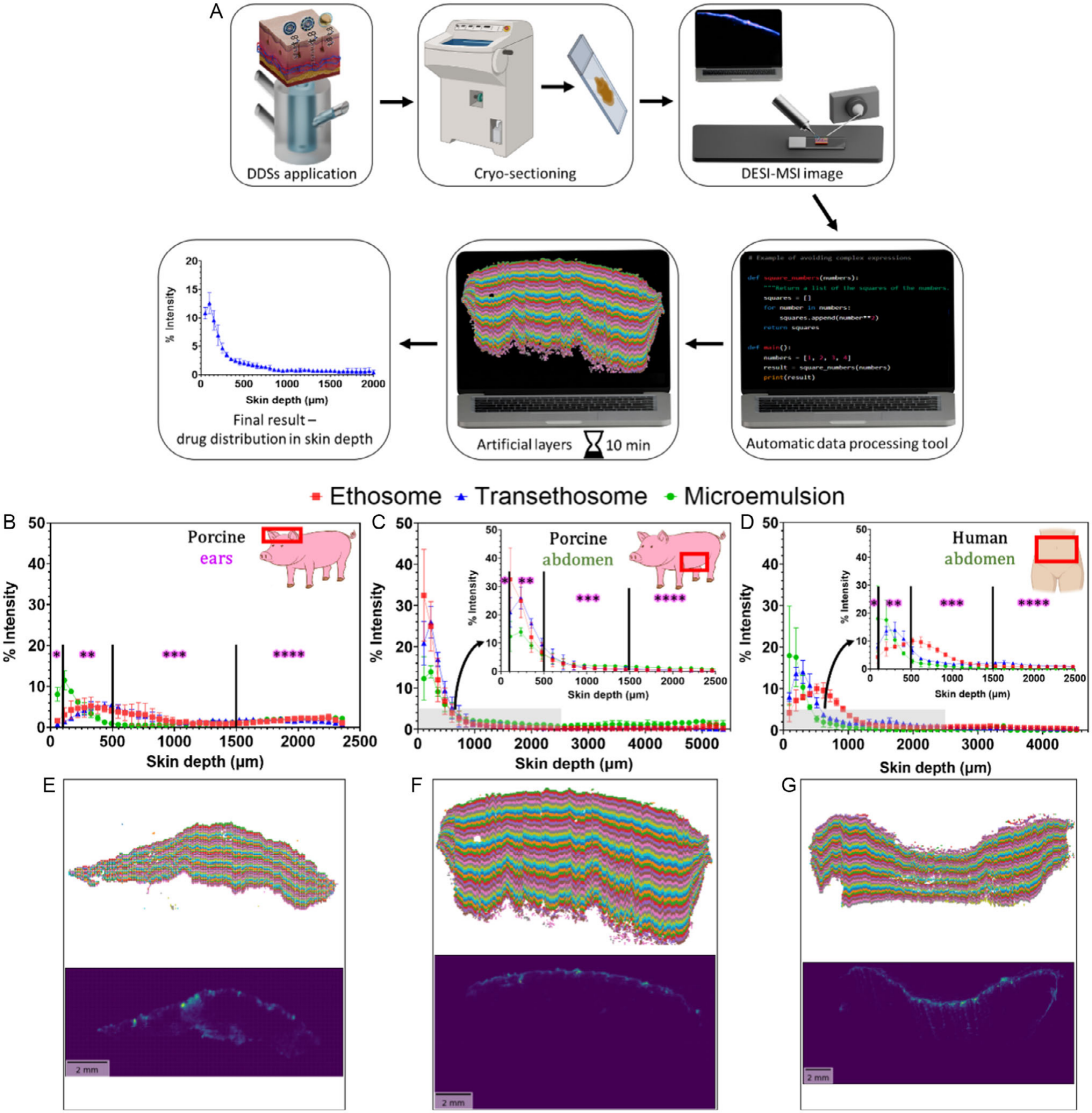
Comparison of TBF skin permeation across three DDSs and three skin sources using the automated tool. A) Flow chart of the experimental process. B–D) Normalized average intensity of TBF signal plotted against skin depth for different skin sources: (B) porcine ear skin, (C) porcine abdominal skin, and (D) human abdominal skin. E–G) Skin is stratified into artificial layers using the automatic tool: (E) porcine ear skin, (F) porcine abdominal skin and G) human abdominal skin. TBF distribution images (*m/z* 292.207) from DESI‐MSI (scale bar: 2 mm) are displayed below each layering image. All images shown are of the ethosomal DDS as an example. All images are oriented with the *SC* at the top; *epidermis, **upper dermis, ***lower dermis, and ****hypodermis.

The automated method was used to compare TBF permeation delivered by different DDSs into skin from various sources (**Figure** **8**A). Results for porcine ear skin (Figure 8B) show that both ethosomal and transethosomal DDSs achieve comparable TBF permeation, reaching depths of up to 1000 μm within 2 h of application. In porcine abdominal skin (Figure 8C), all three DDSs exhibit similar TBF permeation patterns. However, the microemulsion DDS shows slightly lower TBF signal intensity within the first 500 μm compared to the ethosomal and transethosomal DDSs. For human abdominal skin (Figure 8D), the ethosomal DDS achieves greater drug permeation than the transethosomal DDS, reaching depths of up to 1100 μm within 2 h. However, at greater depths (1500–1800 μm), the transethosomal DDS demonstrates a slightly higher TBF intensity signal. A control experiment of TBF skin permeation from an aqueous solution was also performed (Figure S13, Supporting Information), showing an overall inferior permeation profile compared to the DDSs.

These findings suggest that ethosomal and transethosomal DDSs generally provide comparable permeation profiles, effectively delivering TBF to deeper skin layers. However, in skin types with thicker hypodermis, such as porcine abdominal skin, there is no distinct advantage for deep layers delivery of TBF from either DDS, since it's concentrated mostly in the first 500 µm. Conversely, in skin types with thinner fat layers, such as porcine ear and human abdominal skin, the differences in DDS performance are more pronounced. The larger hypodermis layer in porcine abdominal skin may contribute to the lack of variations in TBF permeation rates observed among the DDSs. Stratifying the MS data into thin layers was very fast using the automatic tool ‐ the images shown in Figure 8E–G were created within less than 10 min each. These findings highlight the capability of the automated tool to evaluate DDS permeation across various skin types. This advancement is expected to facilitate the effective design and development of dermal DDSs.

Additionally, we wanted to compare the distribution of TBF and FITC, typically used as its fluorescent permeation marker. For that we added FITC to transethosomal DDS after TBF loading, imaged the distribution pattern of both molecules by DESI‐MSI, and used the automatic method to derive the permeation profiles. Figure S14, Supporting Information shows that FITC and TBF exhibit similar overall permeation patterns, though FITC shows slightly greater permeation at a skin depth of 500–1500 μm. However, it is important to acknowledge that this experiment is not directly comparable to the CLSM study conducted on porcine ear skin (Figure 3) due to anatomical and thickness differences between porcine ear and human abdominal skin. These findings suggest that FITC may serve as a suitable distribution marker for TBF in thicker human skin, although its distribution pattern may differ in thinner skin. Additionally, it should be noted that the permeation profiles of other molecules with differing physicochemical properties may not necessarily align with that of FITC in either skin type.

### 2.8 Kinetic Experiments

**Figure 9:**
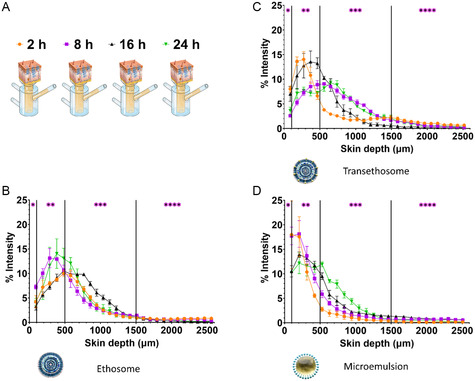
A) Illustration of kinetic experiment. Comparison between three DDSs in different time points (2, 8, 16, and 24 h): B) ethosome, C) transethosome, and D) microemulsion; *epidermis, **upper dermis, ***lower dermis, and ****hypodermis.

The kinetic results presented in **Figure** **9** highlight the impact ‐ of the DDS type on the time‐dependent permeation of TBF into different skin layers. Ethanol‐containing systems (ethosomal and transethosomal DDSs) exhibit rapid TBF release, leading to enhanced permeation into the upper dermis (≈500 μm). This rapid effect is likely due to the potent, yet transient, permeation‐enhancing properties of ethanol, which evaporates quickly after application. As a result, application time has minimal impact on the efficiency of these systems. Notably, transethosomal DDS facilitated deeper delivery (up to ≈1000 μm), while ethosomal DDS favored accumulation in more superficial dermal layers. Transethosomal DDS is an advanced version of the ethosome with reduced size and edge activators for enhanced flexibility, and, as expected, it achieved the deepest skin permeation. In contrast, the ethanol‐free microemulsion demonstrated a more gradual, time‐dependent release profile. With this system, significant TBF delivery to deeper dermal layers was only observed after 24 h, suggesting a sustained release mechanism. These differences highlight the value of kinetic imaging in tailoring DDS selection and application timing to target specific skin depths, an approach particularly relevant for optimizing topical therapies. For example, for treating fungal infections in the lower dermis, TBF delivered via the transethosomal DDS may be the most effective option. Conversely, for infections residing in the epidermis, TBF from the microemulsion DDS may be more suitable, particularly within the first 2 h of application.

## 3 Conclusions

We introduced a detailed methodology for assessing drug distribution across different skin layers following dermal drug delivery. In this methodology, dermal DDSs are first applied to excised skin and equilibrated under physiological conditions, followed by cryo‐sectioning and MSI to visualize drug localization. Precise superimposition of MSI data with histological staining of skin sections enables manual segmentation of skin layers and the comparison of drug ion signal intensity across individual layers. We also report the use of choline as skin tissue marker, which enhances the accuracy of MSI data and histological image alignment. To further streamline data processing, we developed an automated approach for segmenting skin into artificial, size‐controlled layers using only MSI data. This automated method eliminates the need for histological staining and manual pixel‐by‐pixel segmentation, significantly accelerating analysis and reducing processing time from several days to less than 10 min. The algorithm and its user manual are accessible through a web‐based Python platform, as detailed in the SI.

We applied this methodology to three vesicular nanoscale DDSs—ethosomes, transethosomes, and microemulsions—that were designed in this study to deliver the antifungal TBF to specific skin layers. This was performed in three anatomically distinct skin types and allowed detailed characterization of drug delivery mechanisms and kinetics. The study revealed key differences in the performance of these systems: transethosomes demonstrated superior penetration into deeper skin layers, attributed to their enhanced flexibility and deformability; ethosomes showed effective dermal delivery; and the microemulsion system, though initially slower, eventually achieved notable permeation into the lower dermis, suggesting potential for sustained release. These findings emphasize the applicability of this methodology in evaluating not only the depth of drug permeation but also the temporal patterns and mechanisms that govern drug distribution within the skin. Leveraging the significantly reduced data processing times achieved by the automated tool, this approach offers opportunities for studying sublayer‐specific drug distribution within the epidermis, particularly when paired with higher spatial resolution imaging. By advancing the understanding of dermal drug delivery, this methodology holds great promise for guiding the development of targeted and sustained‐release DDSs, addressing diverse therapeutic needs across various skin conditions. These innovations are expected to contribute significantly to precision medicine and the optimization of dermatological therapies.

## 4 Experimental Section

4.1

4.1.1

##### Materials

The following materials were used in this study: MilliQ water—triple deionized water (TDW) (MilliQ, Merck, Germany), methanol absolute ULC‐MS grade (Biolab, Israel), formic acid (Waters Corp., UK), leucine enkephalin (Waters kit), terbinafine hydrochloride (Acros organics, Belgium), ethanol absolute (Carlo Erba reagents, Italy), Lipoid S100 (soybean phosphatidylcholine), phosphatidylcholine content ≥94.0% (Lipoid, Germany), oleic acid (Sigma, Israel), Labrasol (Gattefosse, France), Transcutol (Sigma, Israel), dl‐α‐tocopheryl acetate (Vitamin E acetate) (Pharma‐Tamar, Israel), propylene glycol (Tamar, Israel), cholesterol (Sigma, Israel), M‐1 embedding matrix (Epredia, USA), porcine ear and abdominal skin (Biotech farm LTD, Israel), and human skin (from abdominoplasty surgeries, Hadassah Ein Kerem Medical Center, with Helsinki approval 0731‐20‐HMO). All solvents used were LCMS grade.

##### Methods: DDSs Preparation and Characterization

Ethosomal DDS preparation: Soybean phosphatidylcholine Lipoid S100 (2% w/w) was dissolved in ethanol (50% w/w) for 13 min without mixing. The solution was then mixed with overhead stirrer (Heidolph Hei‐TORQUE 200, Germany) during 1 min at 700 rpm. Propylene glycol (20% w/w) was added through mixing and mixed for 1 min. Vitamin E acetate (0.2% w/w) was then weighted and added with mixing for 1 min. TBF (1% w/w) was then added with mixing. Finally, TDW (26.8% w/w) was added gradually with mixing, and then the final system was mixed for 5 min. 10 mL of the DDS was prepared and had a transparent yellowish appearance.

Transethosomal DDS preparation: Solvent mixture of ethanol (17.9% w/w): propylene glycol (10.1% w/w) 7:3 w/w was prepared. TBF (1% w/w), Lipoid S100 (2% w/w), Tween80 (5% w/w), and cinnamaldehyde (0.3% w/w) were weighted and dissolved in the ethanol: propylene glycol mixture, in a closed vial, stirring with magnetic stirrer bar at 850 rpm. TDW (63.7% w/w) was added slowly to the mixture while stirring, and the final system was stirred for 30 min at 30 °C. Ten mL of the DDS were prepared and had a transparent yellowish appearance.

Microemulsion preparation:^[^ ^56^ ^]^ Oleic acid (5.8% w/w), Labrasol (26.6% w/w), and Transcutol (26.6% w/w) were weighed and mixed together under stirring with magnetic stirrer bar at 1500 rpm. TBF (1% w/w) was added under stirring. After dissolved, TDW (40% w/w) was added under stirring, and the final system was stirred for 20 min. Ten mL of the DDS were prepared and had transparent appearance.

All three systems were characterized by DLS and cryo‐TEM.

The Zetasizer Nano ZSP (Malvern, UK) was employed to assess the size distributions and polydispersity indexes of the DDSs diluted 500 times^[^ ^57^, ^58^, ^59^ ^]^ with TDW at 25 °C. This DLS instrument has a 633 nm laser and utilizes backscattering technology to detect light scattering at 173°. The measurements were performed in triplicate.

The cryo‐TEM imaging was performed using Tecnai 12 G2 Spirit TWIN TEM (ThermoFisher Scientific, USA) equipped with a Gatan 626 cold stage to investigate the system morphology. TIA (Tecnai Imaging & Analysis) software was used to record the images in low dose mode on a 4K × 4K FEI Eagle CCD (charge‐coupled device) camera. Sample preparation was done using Vitrobot Mark IV (ThermoFisher Scientific) as described elsewhere.^[^ ^60^ ^]^

##### Methods: In Vitro Skin Permeation Studies

Skin specimens of full‐thickness human abdomen from surgeries were obtained from abdominoplasty surgeries performed at Hadassah Ein Kerem Medical Center, Jerusalem, Israel (according to ethical requirements with consent from the donors, IRB approval # 0731‐20). Skin specimens of full‐thickness porcine abdominal and porcine ears were purchased from Biotech Farm Ltd., Ramot Meir, Israel. All three skin types were stored at −80 °C prior to use. Human skin was cleaned from fat layer, cut into squares, and stored at −80 °C for later use.

The skin was mounted in Vertical Diffusion Cells (HDT 1000, Copley Scientific Ltd., Nottingham, UK) having cell capacity volume of 12 mL and the skin max exposure area 1.77 cm^2^. TDW was used as the receptor fluid to ensure the skin will not be exposed to unnecessary ions that may interfere with DESI‐MSI measurement. The receptor fluid was mixed using a magnetic stirrer bar at 600 rpm stirring rate, and the temperature was set to reach 32 ± 1 °C on the skin surface.

Ethosomal, transethosomal. and microemulsion DDSs containing 1% of TBF each were evenly applied to the *SC* surface of the skin squares mounted on the diffusion cells, at a dose of 200 μl per skin area, under unoccluded conditions.

Three technical replicates were performed for each of the three skin sources, resulting in nine tissue specimens per timepoint of the study. Additionally, three control permeation experiments were performed: 1) 1% TBF in TDW, 2) 1% FITC added to transethosomal DDS containing TBF, and 3) transethosomal DDS without loaded molecules. In all control experiments, one skin square was used per each control. Treated skin specimens were left on diffusion cells for 2 h for comparison between skin sources, and as continuation experiment for time influence on TBF permeation into human abdominal skin, three timepoints were checked: 8, 16, and 24 h. At each end timepoint, unexposed skin was cut away from the treated areas, and the treated areas were stored in a freezer at −80 °C.

The treated skin specimens were mounted on the cryostat mold using a few drops of M‐1 embedding matrix just for “gluing” the tissue to the surface. M‐1 embedding matrix was chosen because it does not interfere with the MS signal of TBF. The skin specimens were sectioned vertically with the *SC* oriented to the right. This orientation helps avoid potential redistribution of the drug from top to bottom, as well as the spread of lipids from the hypodermis into the dermis. It also reduces the risk of the drug being “pushed” toward the surface during sectioning. The skin sections 20 μm in thickness were created using a Leica CM 1950 Cryostat (Leica Biosystems, USA) with stage temperature of −30 °C and mounted on glass slides (Epredia Netherlands B. V., The Netherlands). Then, the skin sections were stored in a freezer at −80 °C until analysis. Skin sections were vacuum desiccated for 10 min before DESI‐MSI measurement.

##### Methods: DESI‐MSI

The skin sections were scanned by DESI‐MSI (coupled to Xevo G2‐XS QTof mass spectrometer, Waters Corp., UK), which was operated in positive mode at *m/z* 50–1200 scan range and resolving power of 30 000 FWHM. The solvent used was methanol:water:formic acid (98:2:0.01 v/v/v), containing 50 pg μL^−1^ Leucine Enkephalin as internal standard. The solvent flow was 2 μL min^−1^ using a constant flow system (Waters Corp., UK). The constant voltage applied on the solvent was 0.7 kV. Pixel size was 50 μm, and the scan rate was set to 350 μm min^−1^ while the pixel scan time was 0.129 s. The temperature of the heated ion transfer line was set to 30 °C. The final MS image of TBF distribution in the skin was assessed using the HDI software (Waters Corp., UK).

High‐resolution experiments were conducted by DESI‐MSI (coupled to Xevo TQ‐Absolute mass spectrometer, Waters Corp., UK) in positive mode polarity, pixel size 5 μm. MRM transitions were: TBF: 292.2 → 141 collision energy 30 eV, choline: 104.2 → 60 collision energy 15 eV, cone energy 20 V. The pixel scan time was 0.0127 s and the MS rate was 30 scans s^−1^. The solvent used was methanol:water:formic acid (98:2:0.01 v/v/v). The solvent flow was 2 μL min^−1^ using a constant flow system (Waters Corp., UK). The constant voltage applied on the solvent was 0.7 kV. The temperature of the heated ion transfer line was set to 30 °C. The final MS image of TBF distribution in the skin was assessed using the HDI software (Waters Corp., UK).

##### Methods: H&E Staining

The 20 μm thick skin sections were H&E stained immediately after exposure to DESI‐MSI solvent. The H&E staining procedure is detailed in S1. After staining, the slides were let to dry for 1 h under ambient conditions, and then glass coverslip was applied on the slide using Histomount (histological mounting medium, National Diagnostics, USA).

##### Methods: Optical Microscopy

Data were recorded and analyzed using a magnifier digitized with a Motic EasyScan One microscope (Motic China Group Co. Ltd., China) at ×40 magnification (0.26 μm pixel^−1^), using high‐definition scan mode. Motic Easyscan whole‐slide images were viewed with DSAssistant software (Motic China Group Co. Ltd., China).

##### Methods: CLSM

The porcine ear skin specimens were scanned using a CLSM (Zeiss LSM 710 laser scanning microscopy system, Zeiss, Germany) at a stack scanning mode, 2% laser intensity, 10 μm increments through the *z*‐axis with an air plane ×10 objective lens, and 488 nm excitation wavelength. During the microscopic examination, each skin sample was divided into 5 × 5 tiles and micrographic images were obtained. The fluorescence intensity (AUs) was assessed using the ImageJ software.

##### Methods: Algorithm Analysis

MSI data clustering was used twice: first, to separate skin tissue from a background in the DESI‐MSI scan, with a query to user to distinguish between the two. This was tried in several versions to achieve an optimum: using all MS peaks, using a convolutional neural network to choose tissue‐relevant peaks, and using a specific range of peaks. The last one proved the simplest and the most efficient. Second, clustering was done to create equal‐width layers in the tissue cluster (from previous clustering), with the width as user‐determined parameter. Both clustering rounds were done using scikit‐learn Kmeans module.

## Conflict of Interest

The authors declare no conflict of interest.

## Supporting information

Supplementary Material

## Data Availability

The data that support the findings of this study are openly available in [github.com] at [https://github.com/einavres/New_Tissue_Layering_Tool], reference number [1].

## References

[1] A. Zaid Alkilani , M. T. McCrudden , R. F. Donnelly , Pharmaceutics 2015, 7, 438. 26506371 10.3390/pharmaceutics7040438 PMC4695828

[2] K. Parsi , H. Partsch , E. Rabe , A. A. Ramelet , Australas. J. Dermatol. 2011, 52, 159. 21834809 10.1111/j.1440-0960.2011.00749.x

[3] B. Godin , E. Touitou , E. Rubinstein , A. Athamna , M. Athamna , J. Antimicrob. Chemother. 2005, 55, 989. 15857943 10.1093/jac/dki125

[4] T. J. Walsh , D. M. Dixon , Med. Microbiol. 1996, 75, 1.

[5] In Vitro Permeation Test Studies for Topical Drug Products Submitted in ANDAs Guidance for Industry, U.S. department of Health and Human Services Food and Drug Administration and Center for Drug Evaluation and Research (CDER), 2022, Center for Drug Evaluation and Research, Office of Regulatory Policy.

[6] R. Alvarez‐Román , A. Naik , Y. N. Kalia , H. Fessi , R.H. Guy , Eur. J. Pharma. Biopharm. 2004, 58, 301. 10.1016/j.ejpb.2004.03.027 15296957

[7] E. Touitou , H. Natsheh , Pharmaceutics 2021, 13, 2129. 34959410 10.3390/pharmaceutics13122129 PMC8706871

[8] S. Briançon , Eur. J. Dermatol. 2011, 21, 851. 21914580 10.1684/ejd.2011.1494

[9] L. Binder , S. SheikhRezaei , A. Baierl , L. Gruber , M. Wolzt , C. Valenta , J. Dermatol. Sci. 2017, 88, 280. 28826690 10.1016/j.jdermsci.2017.08.002

[10] B. Prideaux , S. J. Atkinson , V. A. Carolan , J. Morton , M. R. Clench , Int. J. Mass Spectrom. 2007, 260, 243.

[11] V. Thiel , P. Sjövall , Time‐Of‐Flight Secondary Ion Mass Spectrometry (TOF‐SIMS): Principles And Practice In The Biogeosciences, The Royal Society of Chemistry, 2014, 4, pp. 122–170.

[12] J. D’Alvise , R. Mortensen , S. H. Hansen , C. Janfelt , Anal. Bioanal. Chem. 2014, 406, 3735. 24722877 10.1007/s00216-014-7802-z

[13] L. S. Eberlin , J. V. Mulcahy , A. Tzabazis , J. Zhang , H. Liu , M. M. Logan , H. J. Roberts , G. K. Lee , D. C. Yeomans , J. Du Bois , J. Am. Chem. Soc. 2014, 136, 6401. 24708172 10.1021/ja501635u PMC4017602

[14] Z. Takats , J. M. Wiseman , R. G. Cooks , J. Mass Spectrom. 2005, 40, 1261. 16237663 10.1002/jms.922

[15] Z. Takats , J. M. Wiseman , B. Gologan , R. G. Cooks , Science 2004, 306, 471. 15486296 10.1126/science.1104404

[16] C. L. Feider , A. Krieger , R. J. DeHoog , L. S. Eberlin , Anal. Chem. 2019, 91, 4266. 30790515 10.1021/acs.analchem.9b00807 PMC7444024

[17] N. A. Belsey , A. Dexter , J.‐L. Vorng , D. Tsikritsis , C. J. Nikula , T. Murta , M.‐V. Tiddia , J. Zhang , E. Gurdak , G. F. Trindade , J. Controlled Release 2023, 364, 79. 10.1016/j.jconrel.2023.10.026 37858627

[18] T. Soudah , A. Zoabi , K. Margulis , Mass Spectrom. Rev. 2023, 42, 751. 34642958 10.1002/mas.21736

[19] K. Margulis , A. S. Chiou , S. Z. Aasi , R. J. Tibshirani , J. Y. Tang , R. N. Zare , Proc. Natl. Acad. Sci. 2018, 115, 6347. 29866838 10.1073/pnas.1803733115 PMC6016785

[20] L. Messer , A. Zoabi , R. Yakobi , H. Natsheh , E. Touitou , K. Margulis , Int. J. Pharm. 2024, 650, 123664. 38061498 10.1016/j.ijpharm.2023.123664

[21] K. Margulis , Z. Zhou , Q. Fang , R. E. Sievers , R. J. Lee , R. N. Zare , Anal. Chem. 2018, 90, 12198. 30188683 10.1021/acs.analchem.8b03410

[22] P. Sjövall , L. Skedung , S. Gregoire , O. Biganska , F. Clément , G. S. Luengo , Sci. Rep. 2018, 8, 16683. 30420715 10.1038/s41598-018-34286-x PMC6232133

[23] D. Bonnel , R. Legouffe , A. H. Eriksson , R. W. Mortensen , F. Pamelard , J. Stauber , K. T. Nielsen , Anal. Bioanal. Chem. 2018, 410, 2815. 29546543 10.1007/s00216-018-0964-3

[24] J. Bunch , M. R. Clench , D. S. Richards , Rapid Commun. Mass Spectrom. 2004, 18, 3051. 15543527 10.1002/rcm.1725

[25] B. Enthaler , J. K. Pruns , S. Wessel , C. Rapp , M. Fischer , K. P. Wittern , Anal. Bioanal. Chem. 2012, 402, 1159. 22139470 10.1007/s00216-011-5562-6

[26] S. Grégoire , G. S. Luengo , P. Hallegot , A.‐M. Pena , X. Chen , T. Bornschlögl , K. F. Chan , I. Pence , P. Obeidy , A. Feizpour , Adv. Drug Delivery Rev. 2020, 153, 137. 10.1016/j.addr.2019.11.004 31778729

[27] A. M. Handler , M. Fallah , A. J. Pedersen , G. P. Pedersen , K. T. Nielsen , C. Janfelt , Int. J. Pharm. 2020, 590, 119949. 33035610 10.1016/j.ijpharm.2020.119949

[28] A. M. Handler , G. P. Pedersen , K. T. Nielsen , C. Janfelt , A. J. Pedersen , M. R. Clench , Eur. J. Pharm. Biopharm. 2021, 159, 1. 33352255 10.1016/j.ejpb.2020.12.008

[29] K. K. Hendel , C. Bagger , U. H. Olesen , C. Janfelt , S. H. Hansen , M. Haedersdal , C. M. Lerche , Drug Delivery 2019, 26, 244. 30859849 10.1080/10717544.2019.1574937 PMC6419659

[30] I. S. Sørensen , C. Janfelt , M. M. B. Nielsen , R. W. Mortensen , N. Ø. Knudsen , A. H. Eriksson , A. J. Pedersen , K. T. Nielsen , Anal. Bioanal. Chem. 2017, 409, 4993. 28687883 10.1007/s00216-017-0443-2

[31] R. Legouffe , O. Jeanneton , M. Gaudin , A. Tomezyk , A. Gerstenberg , M. Dumas , C. Heusèle , D. Bonnel , J. Stauber , S. Schnebert , Anal. Bioanal. Chem. 2022, 414, 5781. 35650447 10.1007/s00216-022-04139-8

[32] X.‐Q. Niu , D.‐P. Zhang , Q. Bian , X.‐F. Feng , H. Li , Y.‐F. Rao , Y.‐M. Shen , F.‐N. Geng , A.‐R. Yuan , X.‐Y. Ying , Int. J. Pharm.: X 2019, 1, 100027. 31517292 10.1016/j.ijpx.2019.100027 PMC6733291

[33] A. Ascenso , S. Raposo , C. Batista , P. Cardoso , T. Mendes , F. G. Praça , M. V. L. B. Bentley , S. Simões , Int. J. Nanomed. 2015, 10, 5837. 10.2147/IJN.S86186 PMC4583114 26425085

[34] A. Kogan , N. Garti , Adv. Colloid Interface Sci. 2006, 123, 369. 16843424 10.1016/j.cis.2006.05.014

[35] D. Paolino , P. Sinha , M. Fresta , M. Ferrari , Encycl. Med. Devices Instrum. 2006.

[36] E. Touitou , B. Godin , C. Weiss , Drug Dev. Res. 2000, 50, 406.

[37] D. Ainbinder , B. Godin , E. Touitou , Percutaneous Penetration Enhancers Chemical Methods In Penetration Enhancement: Nanocarriers, 2016, 4, pp. 61–75.

[38] D. Paolino , G. Lucania , D. Mardente , F. Alhaique , M. Fresta , J. Controlled Release 2005, 106, 99. 10.1016/j.jconrel.2005.04.007 15935505

[39] J. Marto , C. Vitor , A. Guerreiro , C. Severino , C. Eleutério , A. Ascenso , S. Simões , Colloids and Surf. B: Biointerfaces 2016, 146, 616. 27429295 10.1016/j.colsurfb.2016.07.021

[40] D. W. Lachenmeier , J. Occup. Med. Toxicol. 2008, 3, 1. 19014531 10.1186/1745-6673-3-26 PMC2596158

[41] C. K. Song , P. Balakrishnan , C.‐K. Shim , S.‐J. Chung , S. Chong , D.‐D. Kim , Colloids Surf. B: Biointerfaces 2012, 92, 299. 22205066 10.1016/j.colsurfb.2011.12.004

[42] L. B. Lopes , H. VanDeWall , H. T. Li , V. Venugopal , H. K. Li , S. Naydin , J. Hosmer , M. Levendusky , H. Zheng , M. V. L. Bentley , J. Pharm. Sci. 2010, 99, 1346. 19798758 10.1002/jps.21929

[43] P. de la Presa , T. Rueda , M. del Puerto Morales , F. J. Chichón , R. Arranz , J. M. Valpuesta , A. Hernando , J. Phys. Chem. B 2009, 113, 3051. 19708264 10.1021/jp808650e

[44] E. Esposito , M. Drechsler , N. Huang , G. Pavoni , R. Cortesi , D. Santonocito , C. Puglia , Biomed. Microdevices 2016, 18, 1. 27830454 10.1007/s10544-016-0134-3

[45] B. Godin , E. Touitou , Crit. Rev.Ther. Drug Carrier Syst. 2003, 20, 63. 12911264 10.1615/critrevtherdrugcarriersyst.v20.i1.20

[46] Y. Gu , H. Liu , S. Chen , L. Wang , Y. Liu , CIESC J. 2019, 70, 2626.

[47] R. Wong , S. Geyer , W. Weninger , J. C. Guimberteau , J. K. Wong , Exp. Dermatol. 2016, 25, 92. 26284579 10.1111/exd.12832

[48] Y. Wang , R. Xu , W. He , Z. Yao , H. Li , J. Zhou , J. Tan , S. Yang , R. Zhan , G. Luo , Tissue Eng. Part C: Methods 2015, 21, 932. 25781868 10.1089/ten.TEC.2014.0578

[49] E. Touitou , H. Natsheh , J. Zailer , Pharmaceutics 2023, 15, 397. 36839719 10.3390/pharmaceutics15020397 PMC9967029

[50] I. Allon , E. Touitou , Drug Delivery Transl. Res. 2016, 6, 24. 10.1007/s13346-015-0264-9 26644212

[51] T. Subongkot , Drug Delivery 2020, 27, 1087. 32706279 10.1080/10717544.2020.1797244 PMC7470064

[52] J. Zailer , E. Touitou , Drug Delivery Transl. Res. 2014, 4, 416. 10.1007/s13346-014-0204-0 25787204

[53] A. Zeb , O. S. Qureshi , H.‐S. Kim , J.‐H. Cha , H.‐S. Kim , J.‐K. Kim , Int. Journal Nanomed. 2016, 3813. 10.2147/IJN.S109565 PMC4982511 27540293

[54] D. K. Riker , R. H. Roth , X. O. Breakefield and J. Neurochem. 1981, 36, 746. 6257859 10.1111/j.1471-4159.1981.tb01651.x

[55] K. Hoffmann , F. Grafe , W. Wohlrab , R. H. Neubert , M. Brandsch , J. Invest. Dermatol. 2002, 119, 118. 12164933 10.1046/j.1523-1747.2002.01801.x

[56] B. S. Barot , P. B. Parejiya , H. K. Patel , M. C. Gohel , P. K. Shelat , Aaps Pharmscitech 2012, 13, 184. 22187363 10.1208/s12249-011-9742-7 PMC3299475

[57] H. Natsheh , E. Touitou , Drug Delivery Transl. Res. 2018, 8, 806. 10.1007/s13346-018-0503-y 29524165

[58] E. Touitou , H. Natsheh and S. Duchi , Pharmaceutics 2018, 10, 82. 29970859 10.3390/pharmaceutics10030082 PMC6160910

[59] E. Vettorato , M. Fiordelisi , S. Ferro , D. Zanin , E. Franceschinis , G. Marzaro , N. Realdon , Curr. Pharm. Des. 2024, 30, 921. 38482628 10.2174/0113816128289593240226071813

[60] R. Nordström , L. Zhu , J. Härmark , Y. Levi‐Kalisman , E. Koren , Y. Barenholz , G. Levinton , D. Shamrakov , Pharmaceutics 2021, 13, 123. 33478023 10.3390/pharmaceutics13010123 PMC7835975

