# Supplementary material for: A Rapid Assessment Approach for Skin Stratum‐Targeted Drug Delivery Systems Using Mass Spectrometry Imaging and Spatial Clustering
Source: Small Sci. 2025 Jun 1;5(8):2500061. doi: 10.1002/smsc.202500061 (PMC12362830; doi:10.1002/smsc.202500061)
Supplement: Supplementary file 1 — Supplementary Material [file SMSC-5-2500061-s001.pdf]

Supporting Information

A Rapid Assessment Approach for Skin Stratum-Targeted Drug Delivery Systems Using Mass Spectrometry Imaging and Spatial Clustering

Ravit Yakobi Arancibia,[1] Einav Bentov-Arava,[1] Anna Morshin,[1] Jhonathan Elia,[2] Hiba Natsheh,[1] Yael Levi-Kalisman,[3] Rotem Ushki,[1] Anna Elia,[4] Elka Touitou,[1] Katherine Margulis.[1*]

[1] The Institute for Drug Research, the School of Pharmacy, the Faculty of Medicine, The Center for Nanoscience and Nanotechnology, The Hebrew University of Jerusalem, Jerusalem 9112001, Israel.

[2] Department of Plastic Surgery, Hadassah Medical Center, Hebrew University School of Medicine, POB 12000, Jerusalem il-91120, Israel.

[3] Institute of Life Sciences and the Center for Nanoscience and Nanotechnology, The Hebrew University of Jerusalem, Edmond Safra Campus, Givat Ram, Jerusalem 9190401, Israel.

[4] Department of Pathology, Hadassah Medical Center, the Faculty of Medicine, The Hebrew University of Jerusalem, Jerusalem, 91120, Israel.

*Correspondence to: katy.margulis@mail.huji.ac.il

S1. DLS measurements for drug delivery systems

The figure below shows the size distributions and polydispersity indexes (PDI) of three drug delivery systems (DDSs) containing TBF—ethosomes, transethosomes, and microemulsions—characterized by dynamic light scattering (DLS). Measurements were performed in an aqueous phase at a 1:500 dilution, in triplicates. Data were recorded using Zetasizer software (Malvern, UK).

<table>
<tr><td>Ethosome
Average size ± standard deviation (nm)
PdI</td><td>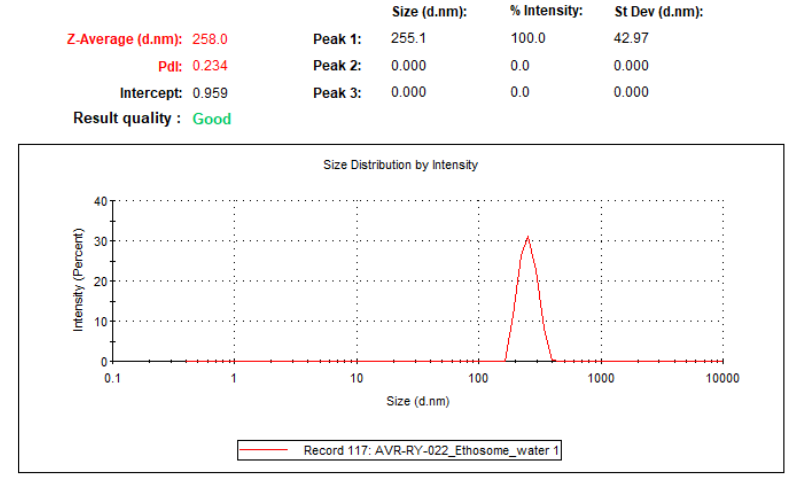

258 ± 43
0.234</td></tr>
<tr><td>Transethosome
Average size ± standard deviation (nm)
PdI</td><td>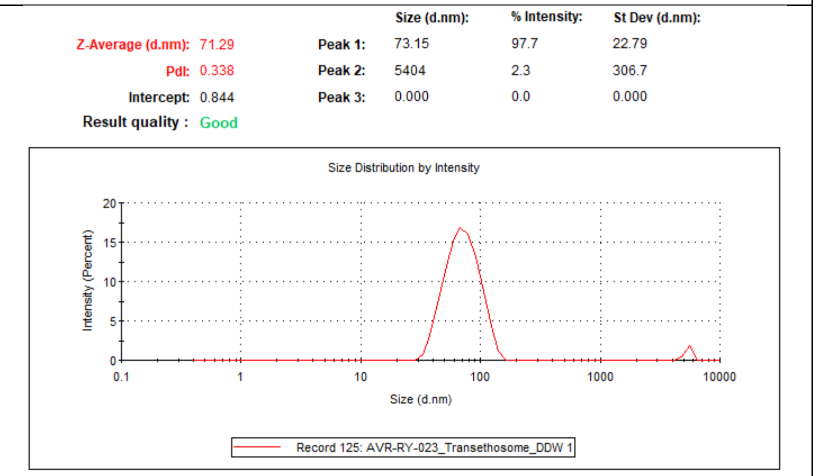

71 ± 23
0.338</td></tr>
<tr><td>Microemulsion
Average size ± standard deviation (nm)
PdI</td><td>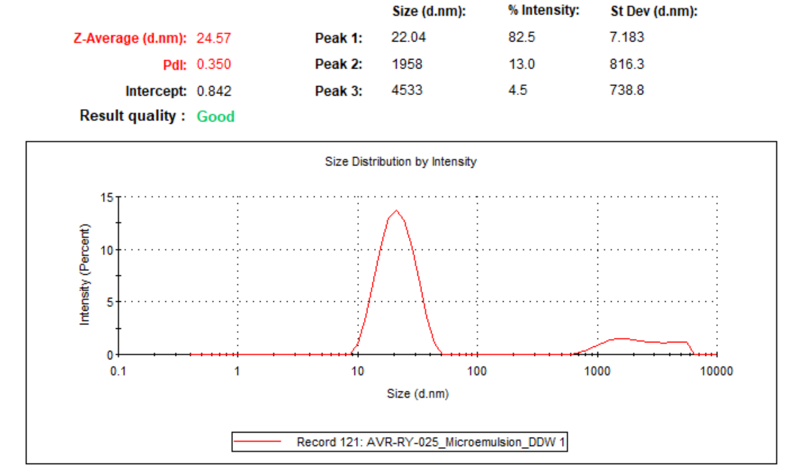

25 ± 7
0.350</td></tr>
</table>

Figure S1. DLS measurements for drug delivery systems

S2. Cryo-TEM analysis for drug delivery systems

The images below depict the structural characterization of three DDSs containing TBF-ethosomes, transethosomes, and microemulsions-using cryo-transmission electron microscopy (cryo-TEM). The scale for each image is indicated below the corresponding figure.

**Ethosome**	**Transethosome**	**Microemulsion**
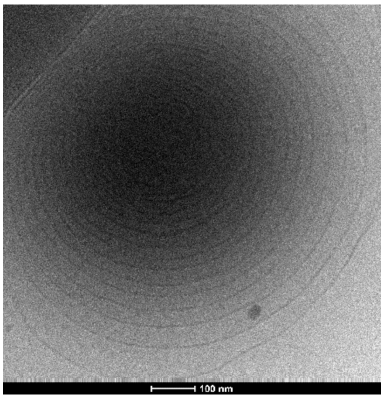	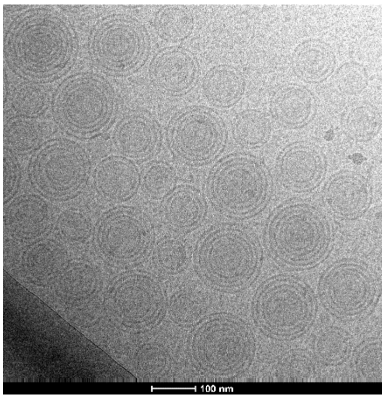	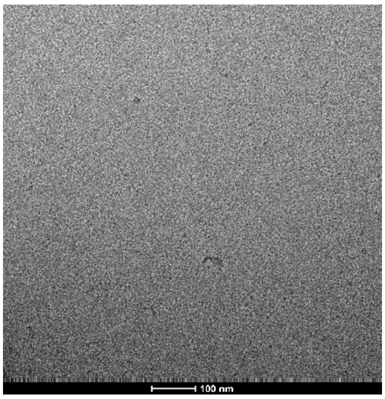
100 nm	100 nm	100 nm
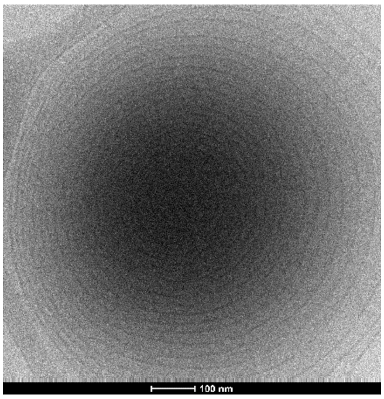	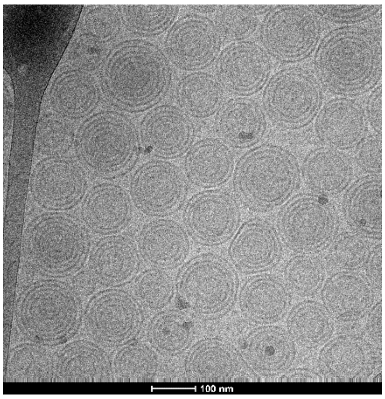	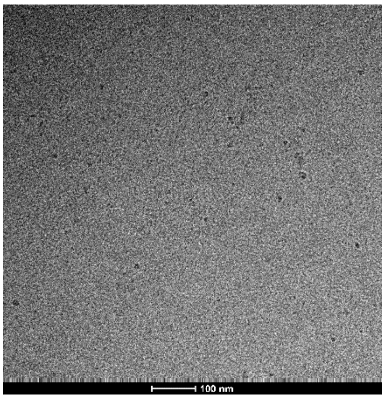
100 nm	100 nm	100 nm
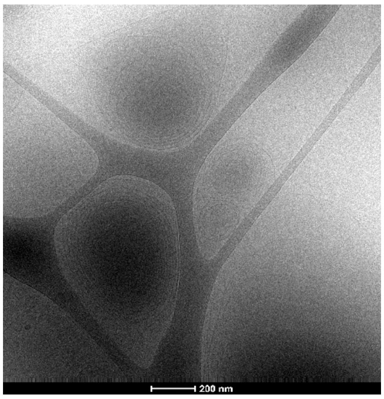	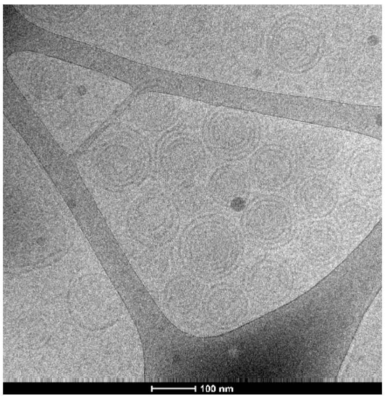	
200 nm	100 nm	
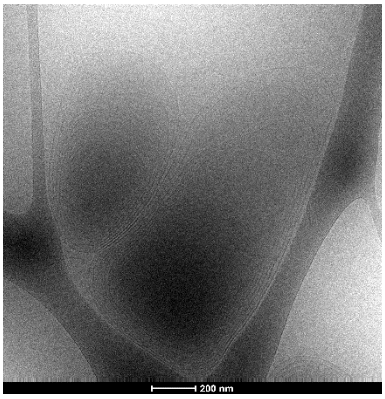	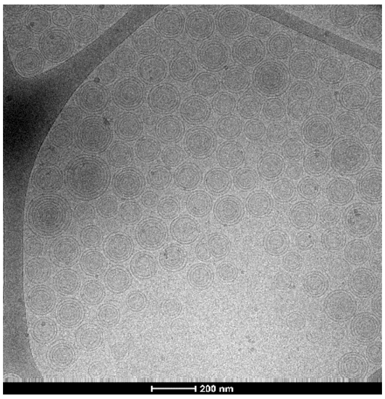	
200 nm	200 nm	

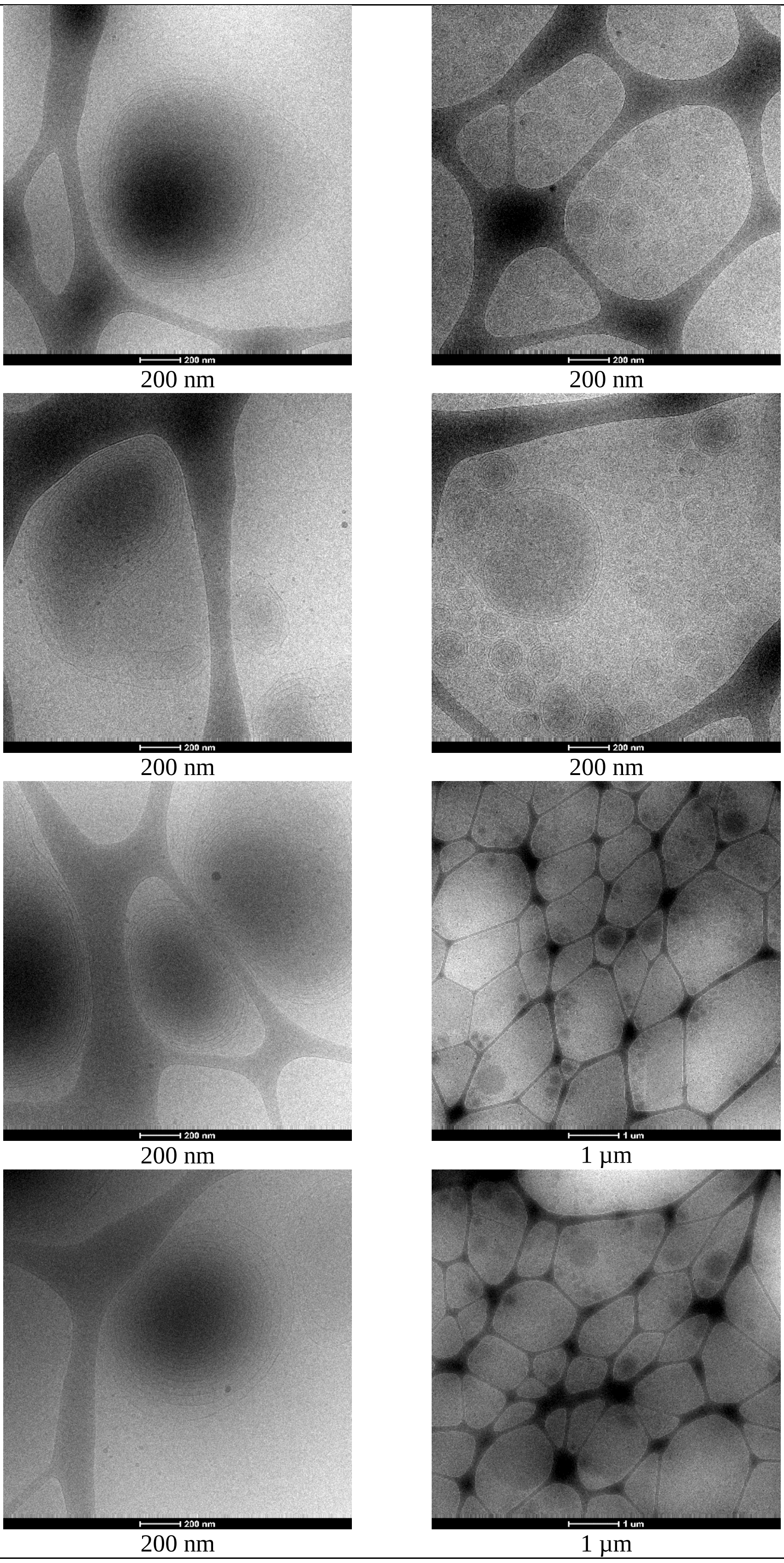

Figure S2. Cryo-TEM analysis for drug delivery systems

S3. Higher resolution (5 μm) imaging

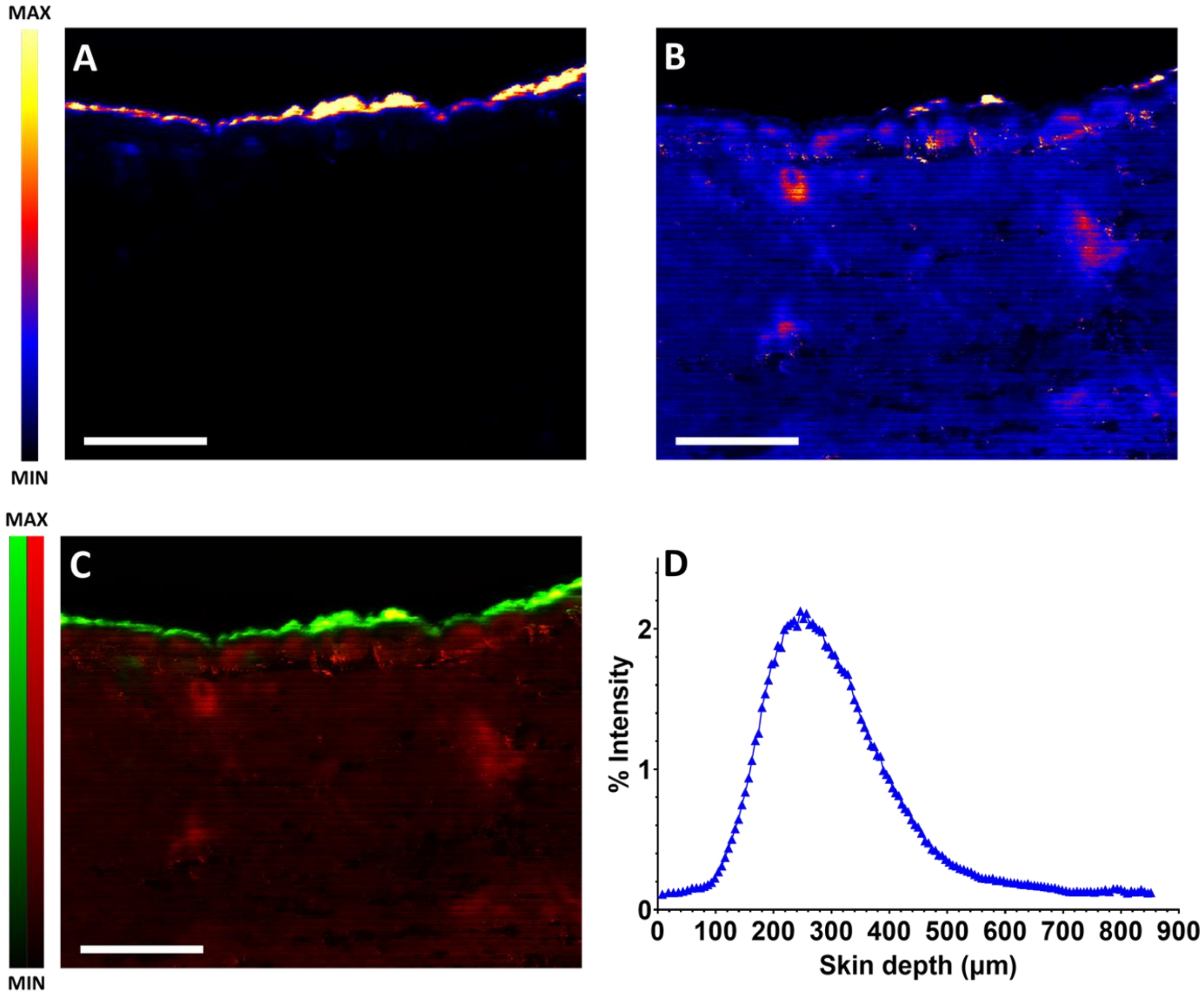

Figure S3. TBF permeation to human abdominal skin from transethosome DDS, using pixel resolution of 5 μm in DESI-MSI. **A.** TBF distribution image *m/z* 292.207. **B.** Choline distribution image *m/z* 104.107. **C.** Overlay between **A** and **B**. Green is TBF and red is Choline. **D.** TBF permeation curve using our automated tool (described in the manuscript section 2.6). Scale bar: 1.5 mm.

S4. TBF signal detection in DESI-MSI

To accurately measure the signal of terbinafine and correct MS spectrum shifts, an internal standard, leucine enkephalin, with a known *m/z*, was added to DESI-MSI solvent. **Figure S4.1** shows the TBF signal detected in the skin tissue sections. The MS spectrum was peak-centered, and the *m/z* of TBF was corrected (**Figure S4.2**). The TBF spectrum from the raw data was then compared to the TBF theoretical spectrum of TBF (**Figure S4.3**). The observed shift between the measured and theoretical spectra was minimal (0.3 ppm), demonstrating the high reliability of the results without requiring molecular fragmentation via the MS/MS method.

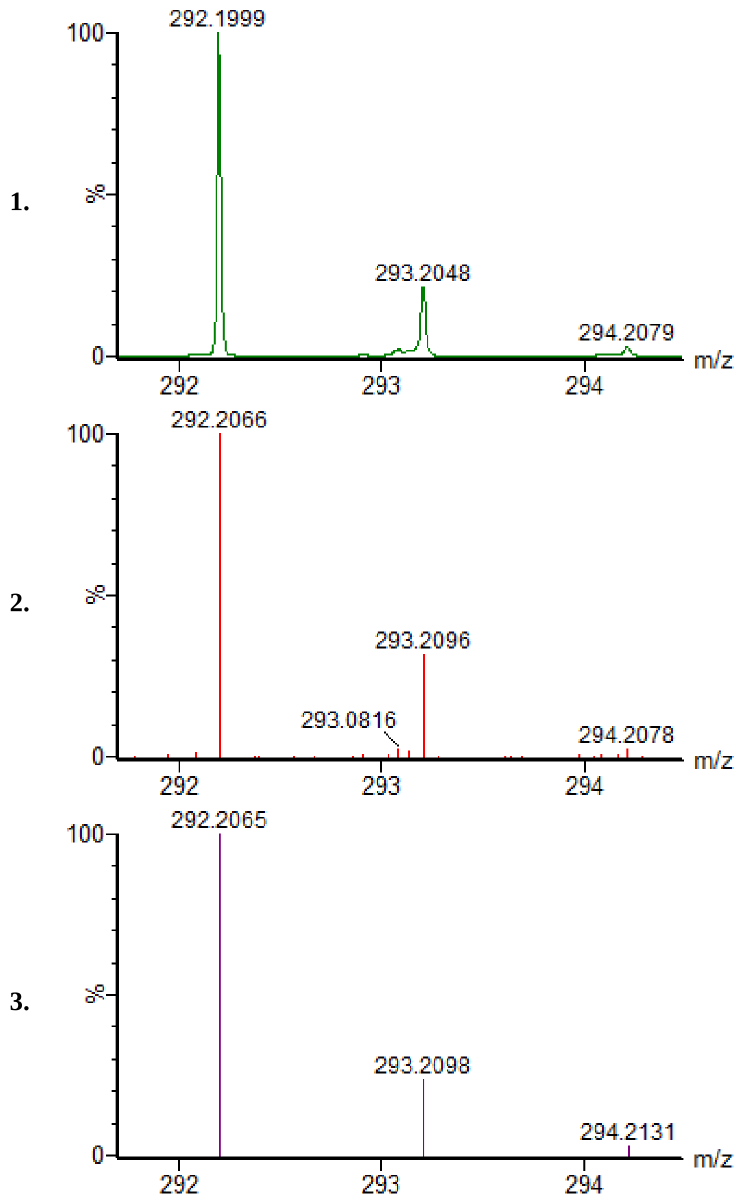

Figure S4. Terbinafine MS spectra (from MassLynx software). **1.** Original data spectrum. **2.** Centered and shift corrected spectrum. **3.** Isotope model of Terbinafine in positive mode ($C_{21}H_{26}N$). Mass error: 0.3 ppm.

S5. Control measurement of skin without TBF

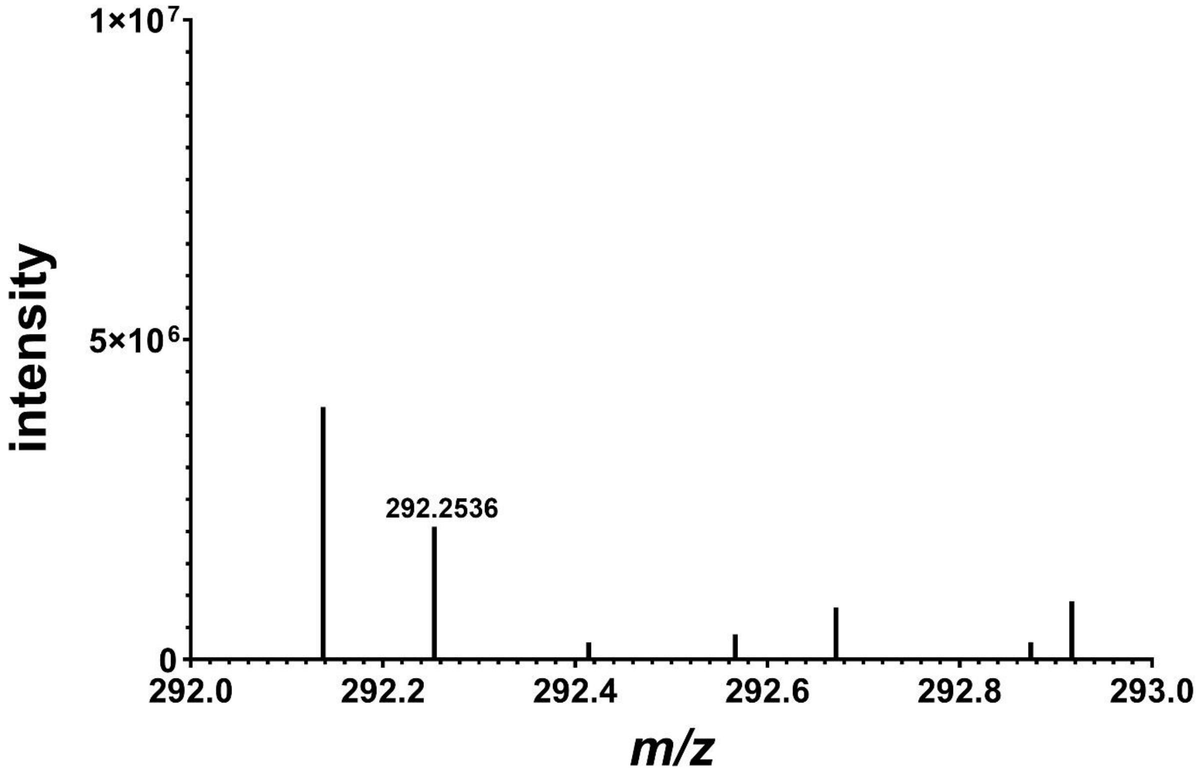

Figure S5. MS spectrum of control skin treated with transethosome DDS without TBF. The *m/z* found is 292.2536, while the *m/z* of TBF is 292.207.

S6. Histological staining

Table S6. Modified H&E staining protocol for unfixed tissues used in this study

Step number	Step description	Step duration
1	Fix in Methanol	2 min
2	Rinse in water	10 dips
3	Stain in Hematoxylin solution	0.5 min
4	Rinse in water	10 dips
5	Blue in 0.1% ammonia	quick dip
6	Rinse in water	10 dips
7	Counterstain in EosinY	0.5 min
8	Rinse and dehydrated in 100% ethanol	10 dips
9	Rinse and dehydrated in 100% ethanol	10 dips
10	Rinse in xylene	6 dips
11	Rinse in xylene	6 dips

S7. Final permeation plot calculation process using the manual method

In this method, the skin tissue is segmented into manually defined regions of interest (ROIs), each represented by a different color and with a width of 50 µm. Using the tissue histology image, the orientation of the skin is determined based on the location of the stratum corneum (*SC*). The average intensity of TBF ion (*m/z* 292.207) within each ROI is extracted using the HDI software. For example, **Figure S7.1** illustrates a porcine ear skin section treated with the ethosomal DDS, explaining the process.

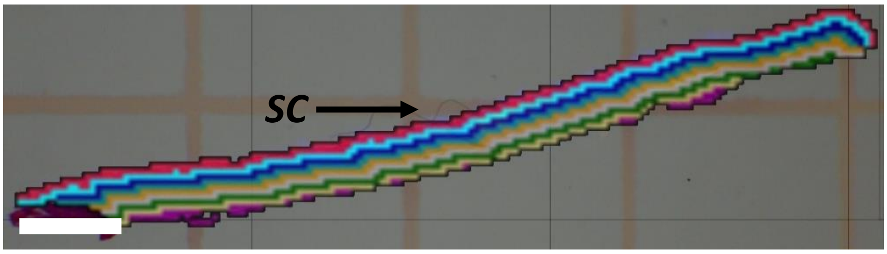

Figure S7.1. Nine ROI layers, represented in different colors, are overlaid on a superimposed image of DESI-MSI and histology, created using HDI software. The first ROI layer, marked in red, corresponds to the *SC*, while subsequent layers represent progressively deeper skin strata. The sample is porcine ear skin treated with the ethosomal DDS. Scale bar: 1 mm.

Table S7.1. Summary of TBF ion average peak intensities in each ROI layer generated by the HDI software. ROI number 1 corresponds to the uppermost layer of the skin containing the *SC*:

ROI	Average intensity of *m/z* 292.207 in ROI
1 (*SC*)	0.00233
2	0.00433
3	0.00324
4	0.00219
5	0.0015
6	0.00086
7	0.00048
8	0.00023
9	0.00007

Table S7.2. The X-axis of the permeation plot represents skin depth, displayed in 50 µm increments corresponding to the width of each ROI layer. The Y-axis, representing the normalized average intensity of TBF (*m/z* 292.207), which is normalized average intensity of TBF (*m/z* 292.207), first the sum of intensities from all layers was calculated (sum=0.01523). Then, each ROI layer normalized intensity was calculated as a fraction of the sum, as shown as an example in layer number 1 (marked in yellow):

X axis: Skin depth (µm)	Y axis: % Intensity of *m/z* 292.207
50	15 (=100*(0.00233/0.01523))
100	28
150	21
200	14
250	10
300	6
350	3
400	2
450	0

The final permeation plot will be:

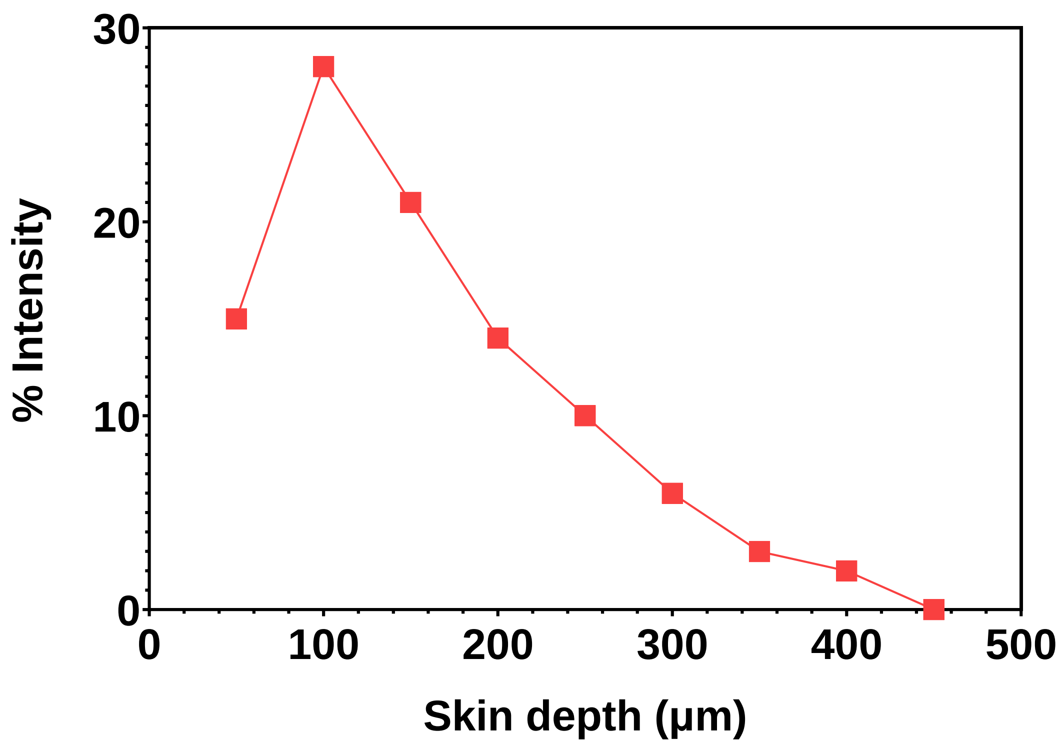

Figure S7.2. Permeation plot of TBF to porcine ear skin using ethosome DDS, calculated manually with ROIs.

S8. Choline signal detection in DESI-MSI

Similar to the TBF signal in the MS spectrum, **Figure S8.1** illustrates the choline signal detected in the skin tissue sections. The MS spectrum was peak-centered and subsequently adjusted using the leucine enkephalin peak to obtain the accurate *m/z* for choline (**Figure S8.2**). The choline spectrum derived from our raw data was then compared to the theoretical spectrum of choline using the isotope model in MassLynx software (**Figure S8.3**). The comparison shows a shift of 1 ppm between the measured data and the theoretical spectrum. While this shift is slightly higher than the error observed with TBF, it is still considered minimal. The increased error in mass accuracy may be attributed to the internal standard *m/z* value (556.2771, $[M+H]^+$), which is closer to the TBF *m/z* than to the choline *m/z*.

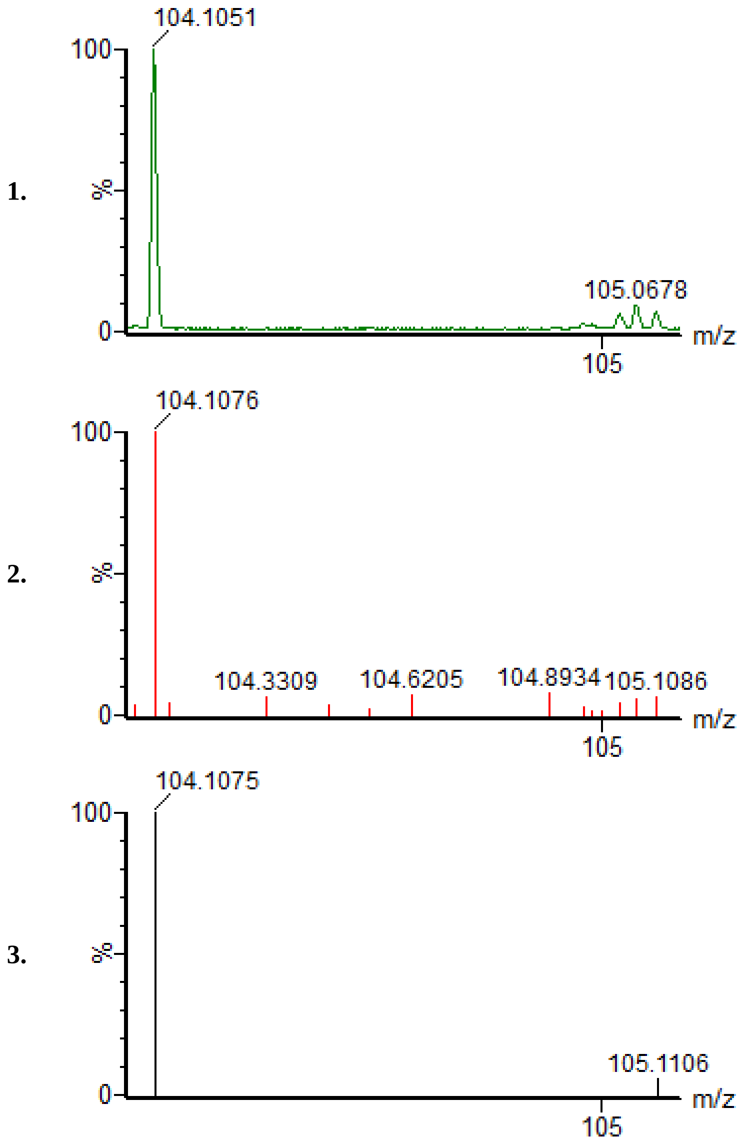

Figure S8. Choline MS spectra (from Masslynx). **1)** Original data spectrum. **2)** Centered and shift corrected spectrum. **3)** Isotope model of Choline in positive mode ($C_5H_{14}NO$). Mass error: 1 ppm.

S9. Manually defined layers on skin tissues

This section presents all images used for the statistical analysis of TBF permeation across three different skin types: porcine ear skin, porcine abdominal skin, and human abdominal skin. Each skin type was tested with three formulations: ethosome, transethosome, and microemulsion. For each formulation, three replicates were analyzed. As this data pertains to the manual calculation method, each tissue section was histologically stained and scanned to enable overlay creation and ROI definition. Below each ROI image, the corresponding histological image of the tissue is provided for reference.

**Ethosome**	**Transethosome**	**Microemulsion**
Porcine ear skin		
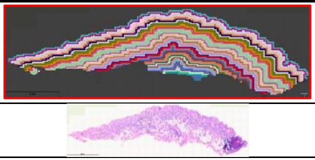	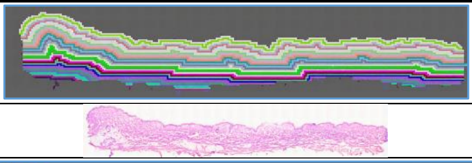	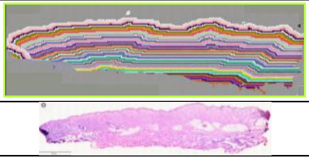
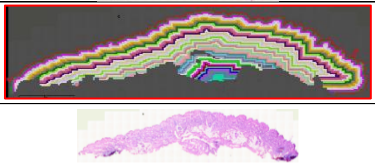	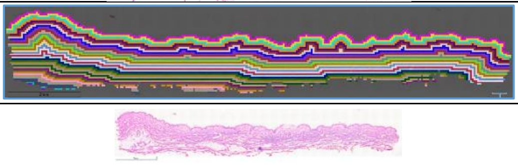	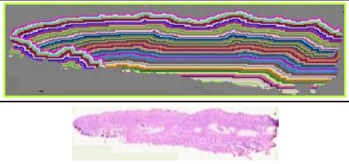
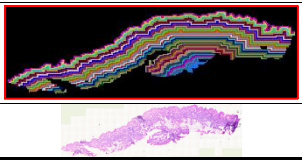	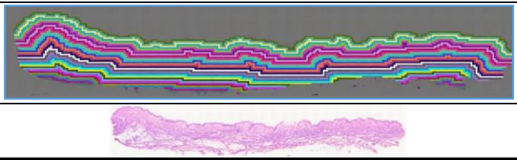	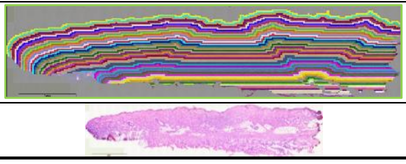
Porcine abdominal skin		
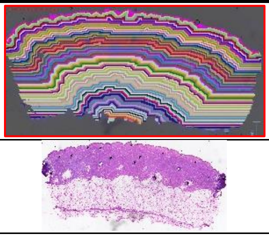	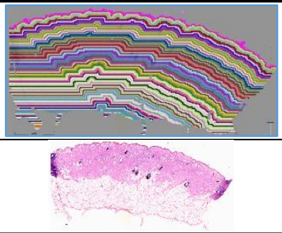	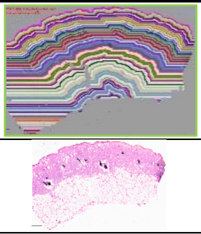
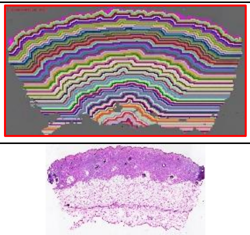	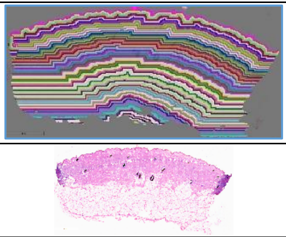	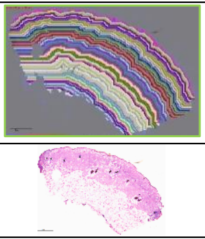
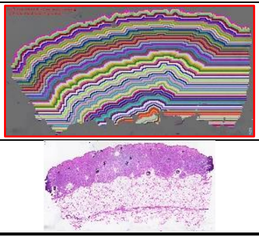	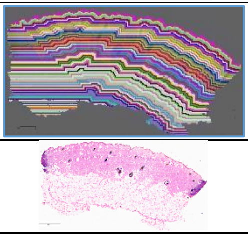	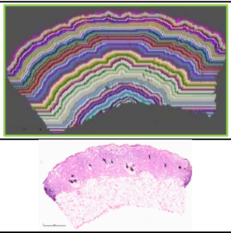
Human abdominal skin		
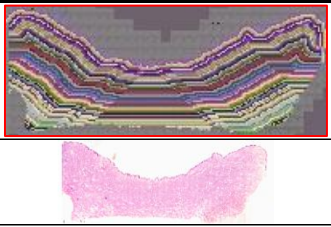	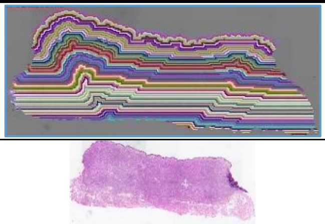	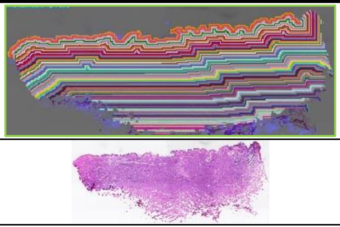

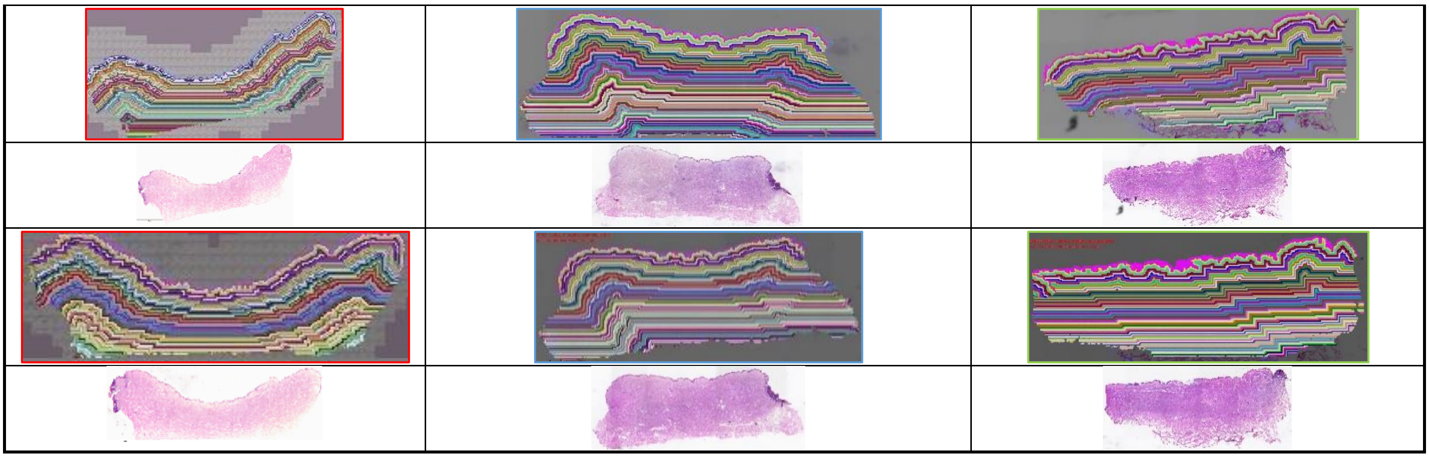

Figure S9. Manually defined layers on skin tissues

S10. User Manual for using the Python Layering tool notebook.

1. Enter to the following link and download the notebook file at: https://github.com/einavres/New_Tissue_Layering_Tool.git. Upload the notebook file to your Google drive, into a specific folder where your data is saved as well as a txt file. This sample txt file is created from the Xevo G2-XS QTof instrument**:**

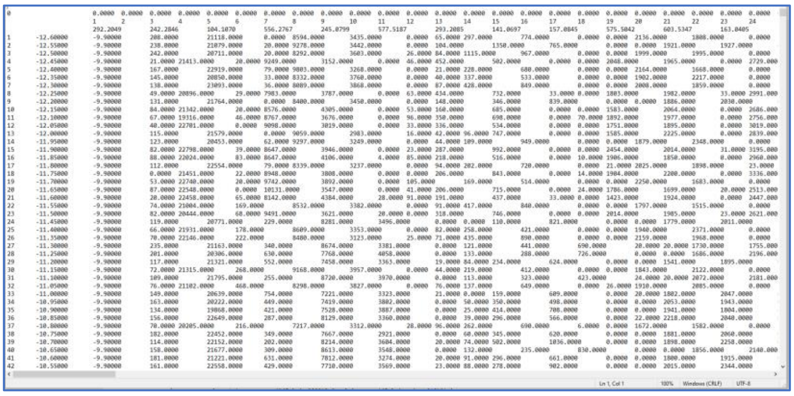

Make sure your txt file looks like in this example. If not, please contact us for adjustments.

2. Open your notebook from your Google drive using google colab app.
3. Connect to hosted runtime: click on the "connect" bottom in the right-up corner. **Colab will need permission to access drive.**
4. Before running the first step of the code, please edit your data file location (marked in yellow). Then, run the first step, clicking on the play icon from the left (marked in yellow):

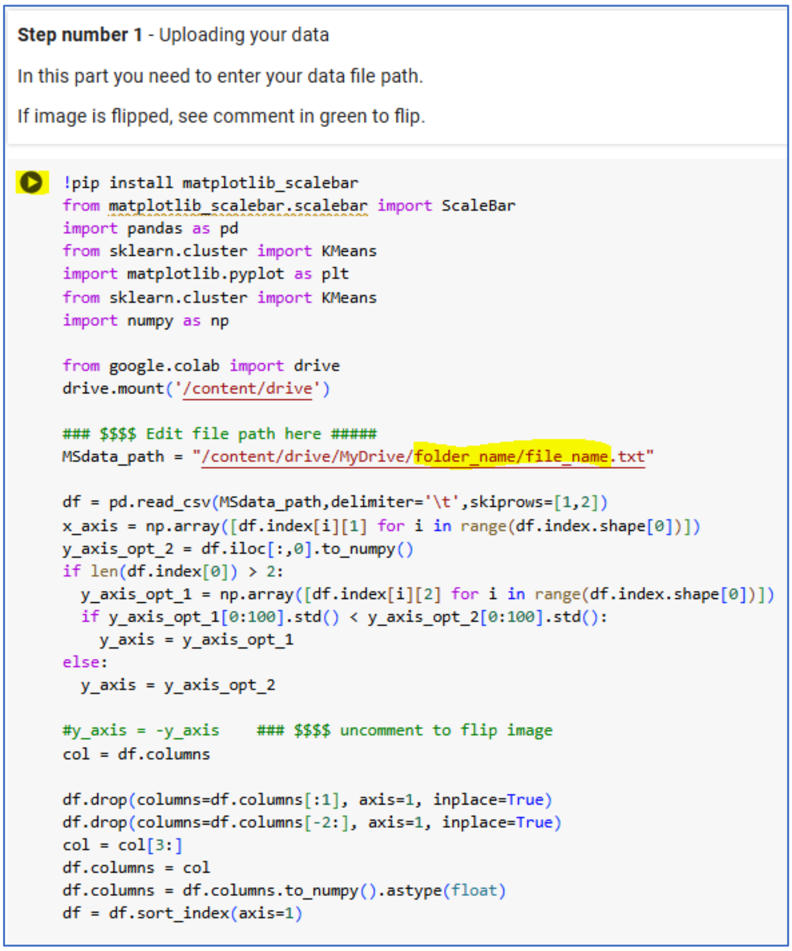

Step number 1 - Uploading your data

In this part you need to enter your data file path.

If image is flipped, see comment in green to flip.

```
!pip install matplotlib_scalebar
from matplotlib_scalebar.scalebar import ScaleBar
import pandas as pd
from sklearn.cluster import KMeans
import matplotlib.pyplot as plt
from sklearn.cluster import KMeans
import numpy as np

from google.colab import drive
drive.mount('/content/drive')

### $$$$ Edit file path here #####
MSdata_path = "/content/drive/MyDrive/folder_name/file_name.txt"

df = pd.read_csv(MSdata_path,delimiter='\t',skiprows=[1,2])
x_axis = np.array([df.index[i][1] for i in range(df.index.shape[0])])
y_axis_opt_2 = df.iloc[:,0].to_numpy()
if len(df.index[0]) > 2:
  y_axis_opt_1 = np.array([df.index[i][2] for i in range(df.index.shape[0])])
  if y_axis_opt_1[0:100].std() < y_axis_opt_2[0:100].std():
    y_axis = y_axis_opt_1
else:
  y_axis = y_axis_opt_2

#y_axis = -y_axis    ### $$$$ uncomment to flip image
col = df.columns

df.drop(columns=df.columns[:1], axis=1, inplace=True)
df.drop(columns=df.columns[-2:], axis=1, inplace=True)
col = col[3:]
df.columns = col
df.columns = df.columns.to_numpy().astype(float)
df = df.sort_index(axis=1)
```

5. Run the second step:

Step number 2 - Introducing your data peaks

```
df.columns
```

```
Index([  74.063,  84.9628, 100.0785, 103.9805,  104.107, 105.1122, 115.0547,
       116.9882,  118.088, 129.0542, 130.0883, 132.0784,   140.07, 141.0697,
       142.0744, 148.0989, 149.0245, 155.0701, 157.0845, 158.0153, 163.0405,
       169.0862, 170.0828, 172.0976, 173.0811, 177.0568, 177.0912, 183.0771,
        185.118, 191.1068, 197.0886,  203.056, 217.0429, 217.1079,  223.098,
       225.0958,  227.127, 229.1442, 239.1641, 242.2846, 243.2897, 245.0799,
       246.0842,  249.149, 269.2113,  279.102, 292.2049, 293.2085, 297.6152,
       299.1137, 301.1433, 308.2025, 353.2674,  413.269, 447.3492, 448.3532,
       467.1055, 467.3745, 469.3308, 470.3352, 493.3917, 506.4216, 507.4113,
       536.1682, 549.4893, 551.5047, 556.2767, 557.2819, 558.2871, 573.4892,
       575.5042, 576.5081, 577.5187, 578.2606, 578.5234,  579.264, 579.5339,
       580.5388, 589.4902, 591.5012, 592.5048, 593.5147,  594.227, 594.5195,
       599.3976, 599.5047,  600.243, 601.5195, 602.5239, 603.5347, 604.5389,
       605.5488,  606.553, 610.1878, 615.4985, 617.5144, 618.5183, 619.5292,
       620.5336, 633.5003],
      dtype='float64')
```

6. Find your peak of interest from the list of peaks and insert its number in the target peak definition (marked in yellow) in step number 3, and then run:

Step number 3 - Insert your peak of interest

Find your peak from the list in Step number 2 and insert it instead of the existing number.

```
#### find your peak $$$$
target_peak = 292.2049
```

7. Run step number 4:

Step number 4 - View peak distribution image

1 pixel = 50 micrometer. This parameter depends on your instrument settings.

```
aspect_ratio = 1
plt.axes().set_aspect(aspect_ratio, 'box')
plt.xlim(min(x_axis), max(x_axis))
plt.ylim(min(y_axis), max(y_axis))
plt.scatter(x_axis.T, y_axis.T, marker='.', c = df[target_peak],s = 3)
plt.title('peak '+str(target_peak) + " " )
scalebar = ScaleBar(1,'mm',box_alpha=0.5,location="lower left",    rotation="horizontal")
plt.gca().add_artist(scalebar)
plt.show()
```

8. Run step number 5:

Step number 5 - Normalization of data

Different types of normalizations are offered (assigned in green): normalization by maximum intensity (max-norm), normalization by mean intensity (mean-norm), and normalization by the sum of the intensities (sum-norm) also known as TIC (total ion current).

Those normalizations can be done per pixel or per row.

Please choose the one is best for your data.

```
#### without normalization
df_copy = df.copy()
plt.axes().set_aspect(aspect_ratio, 'box')
plt.scatter(x_axis.T, y_axis.T, marker='.', c = df_copy[target_peak],s = 3)
plt.show()

#### check if normalization is needed

#### Pixel-wise normalization
#### max-norm
df_copy = df.copy()
df_copy = df_copy.div(df.max(axis=1)+0.0000001 ,axis = 0)
plt.axes().set_aspect(aspect_ratio, 'box')
plt.scatter(x_axis.T, y_axis.T, marker='.', c = df_copy[target_peak],s = 3)
plt.show()

#### mean-norm
df_copy = df.copy()
df_copy = df_copy.div(df.mean(axis=1)+0.0000001 ,axis = 0)
plt.axes().set_aspect(aspect_ratio, 'box')
plt.scatter(x_axis.T, y_axis.T, marker='.', c = df_copy[target_peak],s = 3)
plt.show()

#### sum-norm (TIC)
df_copy = df.copy()
df_copy = df_copy.div(df.sum(axis=1)+0.0000001 ,axis = 0)
plt.axes().set_aspect(aspect_ratio, 'box')
plt.scatter(x_axis.T, y_axis.T, marker='.', c = df_copy[target_peak],s = 3)
plt.show()
```

9. Run step number 6. Note that you can change the number of clusters (marked in yellow), in case the clustering does not suffice:

Step number 6 - Data clusters

In this part the image is divided into clusters based on the MS data.

You need to choose the number of clusters (K parameter) suitable for your image in order to distinguish between background and tissue.

```
import matplotlib.colors as mcolors

df_norm = df.copy()

### add normalization if needed:

# df_norm = df.div(df.mean(axis=1)+0.0000001 ,axis = 0)  #### mean per pixel norm
# df_norm = df.div(df.sum(axis=1)+0.0000001 ,axis = 0)  ####TIC per pixel norm
# df_norm = df.div(df.max(axis=1)+0.0000001 ,axis = 0) ### max per pixel norm

# for y in np.unique(y_axis):    ### mean per row normalization
#    df_norm.iloc[y_axis == y] = df_norm.iloc[y_axis == y].div(df_norm.iloc[y_axis == y].mean())

# for y in np.unique(y_axis):   #### max per row normalization
#    df_norm.iloc[y_axis == y] = df_norm.iloc[y_axis == y].div(df_norm.iloc[y_axis == y].max())

# for y in np.unique(y_axis):  #### sum-norm (TIC) per row normalization
#    df_norm.iloc[y_axis == y] = df_norm.iloc[y_axis == y].div(df_norm.iloc[y_axis == y].sum())


plt.axes().set_aspect(aspect_ratio, 'box')
plt.scatter(x_axis.T, y_axis.T, marker='.', c = df_norm.mean(axis = 1),s = 3)


data_mesh = df_norm.to_numpy()
C = list(mcolors.TABLEAU_COLORS)
K = 17                    #### choose number of clusters
km = KMeans(n_clusters=K)

km.fit(data_mesh)
c_ = km.predict(data_mesh)
plt.axes().set_aspect(aspect_ratio, 'box')
```

10. Before running step number 7, choose the background clusters from step number 6 and insert them into the yellow marked area:

Step number 7 - Background clusters

In this part you need to insert the cluster numbers that match background as it shown in the results of Step number 6.

```
### $$$ pick background clusters  ###

bg_clusters =[9,10] #### insert the cluster numbers that match background, separated by ","

plt.axes().set_aspect(aspect_ratio, 'box')
plt.xlim(min(x_axis), max(x_axis))
plt.ylim(min(y_axis), max(y_axis))
for k in bg_clusters:
  my_members = (c_ == k)

  plt.scatter(x_axis[my_members].T, y_axis[my_members].T, marker='.',c=C[k%9],s = 3)

plt.title('background layout '  )
plt.show()
```

11. Run step number 8, make sure tissue is captured correctly:

Step number 8 - Show final tissue cluster

```
### Show final tissue ###
tissue_cluster_pix = [pix not in bg_clusters for pix in c_]
tissue = df_norm[tissue_cluster_pix]
data_mesh_tissue = tissue.to_numpy()
plt.axes().set_aspect(aspect_ratio, 'box')
plt.xlim(min(x_axis), max(x_axis))
plt.ylim(min(y_axis), max(y_axis))
plt.scatter(x_axis[tissue_cluster_pix].T, y_axis[tissue_cluster_pix].T, marker='.',c=C[k%9],s = 3)
plt.title('tissue layout '  )
plt.show()

df_norm['x'] = x_axis
df_norm['y']  = y_axis
tissue = df_norm[tissue_cluster_pix]
x_axis_tissue = tissue['x']
y_axis_tissue = tissue['y']
```

12. If cropping of the image is needed, remove the comment sign from all lines in step number 9 and edit axis X and\or Y as described below, and then run step number 9:

Step number 9 - Final tissue image cropping

If cropping of the final tissue image is needed, please remove the commnet sign in all lines, and edit the second line for the y or/and x axis cropping.

```
# #### optional - if cropping needed
# tissue_cluster_pix = np.logical_and( tissue_cluster_pix ,df_norm['y']<9 )   #### change condition here: x/y , >/< , threshold number
# tissue = df_norm[tissue_cluster_pix]
# data_mesh_tissue = tissue.to_numpy()
# plt.axes().set_aspect(aspect_ratio, 'box')
# plt.xlim(min(x_axis), max(x_axis))
# plt.ylim(min(y_axis), max(y_axis))
# plt.scatter(x_axis[tissue_cluster_pix].T, y_axis[tissue_cluster_pix].T, marker='.',s = 3)
```

13. Run step number 10:

Step number 10 - Straightening the tissue

```
str_tissue = tissue.copy()
str_tissue['y'] = tissue.groupby('x')['y'].transform(lambda x: ((x- x.mean())/x.std()))
str_tissue['y']  = str_tissue['y']*100000
str_tissue_np = str_tissue.to_numpy()
plt.scatter(str_tissue_np[:,-2].T, str_tissue_np[:, -1].T, marker='.',c=C[k%9],s = 3)
plt.title('straight tissue' )
plt.show()
```

14. Before running step number 11, insert in the K parameter (marked in yellow) the number of layers you wish to divide your tissue to, and then run step number 11:

Step number 11 - Divide the tissue to layers

In this part you need to choose the number of layers you want to divide your tissue to (K parameter).

As the K number is higher, the layers will be thinner.

```
#### make layers

K = 50  ### $$$$$ for thinner layers up this number

str_tissue_np_xy = str_tissue_np[:,[-1,-2]]
str_tissue_np_xy = np.nan_to_num(str_tissue_np_xy)

km = KMeans(n_clusters=K)
km.fit(str_tissue_np_xy)
c_ = km.predict(str_tissue_np_xy)


df_results_new = pd.DataFrame()
for k in range(K):
  my_members_thin = (c_ == k)
  str_tissue_np
  df_results_new.loc[k,'h'] =  str_tissue_np_xy[my_members_thin].mean()
```

15. Run step number 12:

Step number 12 - Final tissue divided into layers

```
plt.figure(figsize=(20,12))
plt.axes().set_aspect(aspect_ratio, 'box')
plt.xlim(min(x_axis), max(x_axis))
plt.ylim(min(y_axis), max(y_axis))

i = 0
for k in K_re:
  my_members_thin = (c_ == k)
  plt.scatter(x_axis_tissue[my_members_thin].T, y_axis_tissue[my_members_thin].T, marker='.',c=C[k%9],s = 22)
  i = i+1

plt.show()
```

16. Run step number 13:

Step number 13 - Show each layer in separate image

```
i = 0
for k in K_re:
  plt.clf()
  my_members_thin = (c_ == k)
  plt.scatter(x_axis_tissue[my_members_thin].T, y_axis_tissue[my_members_thin].T, marker='.',c=C[k%9],s = 22)
  plt.title('KMeans_apart_'+str(i) + " " + str(C[i%10] +" "+ str(int(i/10))) + "    old:"+ str(k) )
  i=i+1
  plt.show()
```

17. Run step number 14:

Step number 14 - Create a table with final results

In this part you can see in a table the information about your peak of interest intensity in each layer.

You will receive the mean width of pixels in each layer. To convert it to meters units you need to multiplicate with your pixel size in meters.

```
#### creates results csv file with table

df_results = pd.DataFrame()
i = 0
for k in K_re:
  df_results.loc[k,'color+id'] = str(C[i%10] +" "+ str(int(i/10)))
  thin_cluster_pix = (c_ == k)
  df_results.loc[k,'y_max'] = tissue[thin_cluster_pix]['y'].max()
  df_results.loc[k,str(target_peak) +" mean intensity"] = tissue[thin_cluster_pix][target_peak].mean()
  df_results.loc[k,'mean width (pixels)'] = tissue[thin_cluster_pix].groupby('x')['y'].count().mean()
  i=i+1

sum_target = df_results.loc[:,str(target_peak) +" mean intensity"].sum()
df_results.reset_index(drop = True, inplace = True)
df_results.loc[:,'normalzed_target'] = df_results.loc[k,str(target_peak) +" mean intensity"]/sum_target


df_results.tail(25)
```

18. Before running step number 15, edit the path for saving your final excel file, and then run step number 15:

Step number 15 - Create excel file with the results

In this part you need to change the file path where you want your results to be saved.

```
### $$$$ change path name before saving

path ="/content/drive/MyDrive/folder_name/Results_file_name.csv"
df_results.to_csv(path)
```

S11. Clustering method optimization

Several methods were initially explored for clustering MSI data. The first approach involved a simple attempt to divide the signal into two clusters: tissue and background ROIs. However, this method proved inadequate, as some tissue regions, particularly from the uppermost layers, were misclassified due to significant intra-tissue signal variations. This highlighted the need for a more precise clustering approach to improve accuracy.

Next, clustering was performed using signal data in combination with spatial location, aiming for more spatially consistent results. Ultimately, the best outcomes were achieved by employing a higher-order clustering method, guided by user input to accurately differentiate tissue from the background. This approach also included cropping the image to eliminate outliers and further enhance accuracy.

S12. Final permeation calculations process (automated method)

The skin tissue is divided into artificial layers, each one presented in different color.

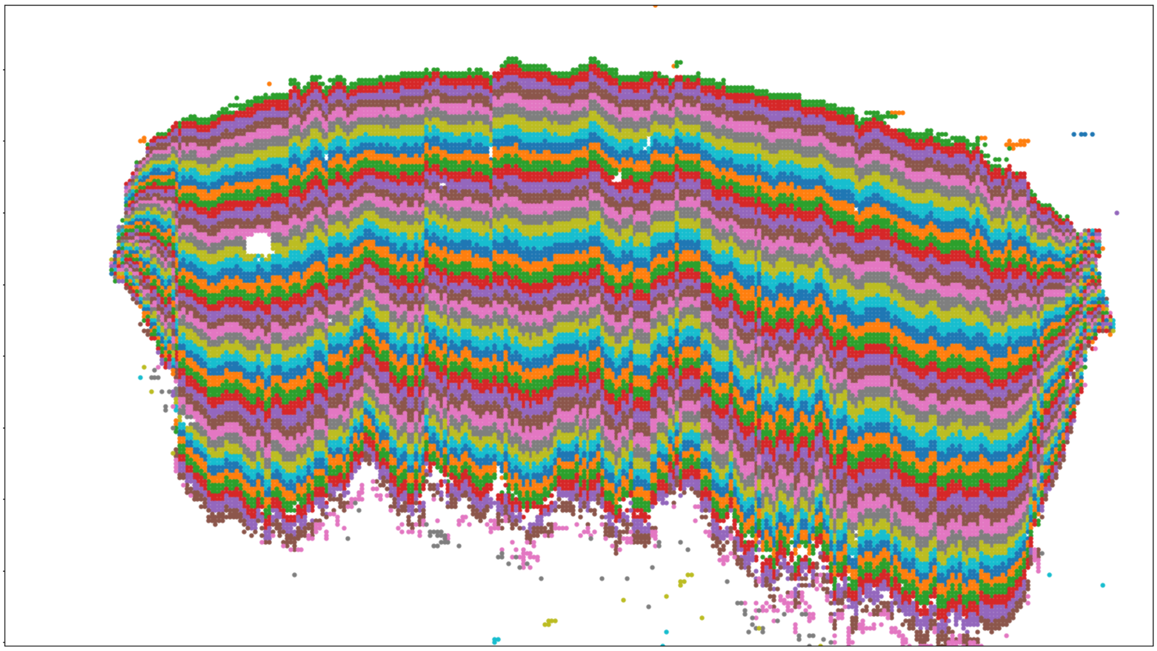

Figure S12.1. Human abdominal skin treated with the ethosomal DDS. This image shows the division of the skin into 45 artificial layers, using the Python code.

Table S12.1. The final step of the code produced an Excel file containing the following data, where layer number 1 corresponds to the uppermost layer of the skin:

Layer number	mean width (pixels)	m/z 292.207 mean intensity
1	2	101372
2	2	166345
3	2	205985
4	2	222487
5	2	194115
6	2	125016
7	2	85195
8	2	48059
9	2	26900
10	2	25056
11	2	16283
12	2	8344
13	2	4991
14	2	6039
15	2	7171
16	2	3309
17	2	3482
18	2	2536
19	2	2626
20	2	1442
21	2	1486
22	2	1246
23	2	1390
24	2	998
25	2	702
26	2	562
27	2	650
28	2	640
29	2	863
30	2	982
31	2	616
32	2	494
33	2	491
34	2	476
35	2	458
36	2	372
37	2	378
38	2	336
39	2	274
40	2	317
41	2	333
42	2	321
43	2	303
44	2	264
45	1	187

Table S12.2. To calculate the X-axis representing skin depth in µm, the mean width of the first layer in pixels was multiplied by 50 (as each pixel represents 50 µm). This calculation is shown for layer 1 (highlighted in yellow). Subsequently, the same calculation was performed for each additional layer, cumulatively adding the width of the upper layers in µm. Layers 2 and 3 provide examples of this calculation (highlighted in yellow).

Layer number	Mean width (pixels)	Skin depth (µm) (X axis)
**1**	**2**	**100 (50*2)**
2	2	200 (100+50*2)
3	2	300 (200+50*2)
4	2	400
5	2	500
6	2	600
7	2	700
8	2	800
9	2	900
10	2	1000
11	2	1100
12	2	1200
13	2	1300
14	2	1400
15	2	1500
16	2	1600
17	2	1700
18	2	1800
19	2	1900
20	2	2000
21	2	2100
22	2	2200
23	2	2300
24	2	2400
25	2	2500
26	2	2600
27	2	2700
28	2	2800
29	2	2900
30	2	3000
31	2	3100
32	2	3200
33	2	3300
34	2	3400
35	2	3500
36	2	3600
37	2	3700
38	2	3800
39	2	3900
40	2	4000
41	2	4100
42	2	4200
43	2	4300
44	2	4400
45	1	4500

Table S12.3. The Y-axis, representing the %intensity of the drug peak (*m/z* 292.207), was calculated by first determining the sum of intensities across all layers (sum = 1271892). Each layer's normalized intensity was then computed as a fraction of sum, multiplied by 100. Layer 1 provides an example of this calculation (highlighted in yellow).

Layer number	m/z 292.207 mean intensity	% Intensity (Y axis)
1	101372	8.0 (100*(101372/1271892))
2	166345	13.1
3	205985	16.2
4	222487	17.5
5	194115	15.3
6	125016	9.8
7	85195	6.7
8	48059	3.8
9	26900	2.1
10	25056	2.0
11	16283	1.3
12	8344	0.7
13	4991	0.4
14	6039	0.5
15	7171	0.6
16	3309	0.3
17	3482	0.3
18	2536	0.2
19	2626	0.2
20	1442	0.1
21	1486	0.1
22	1246	0.1
23	1390	0.1
24	998	0.1
25	702	0.1
26	562	0.0
27	650	0.1
28	640	0.1
29	863	0.1
30	982	0.1
31	616	0.0
32	494	0.0
33	491	0.0
34	476	0.0
35	458	0.0
36	372	0.0
37	378	0.0
38	336	0.0
39	274	0.0
40	317	0.0
41	333	0.0
42	321	0.0
43	303	0.0
44	264	0.0
45	187	0.0

The final permeation plot is:

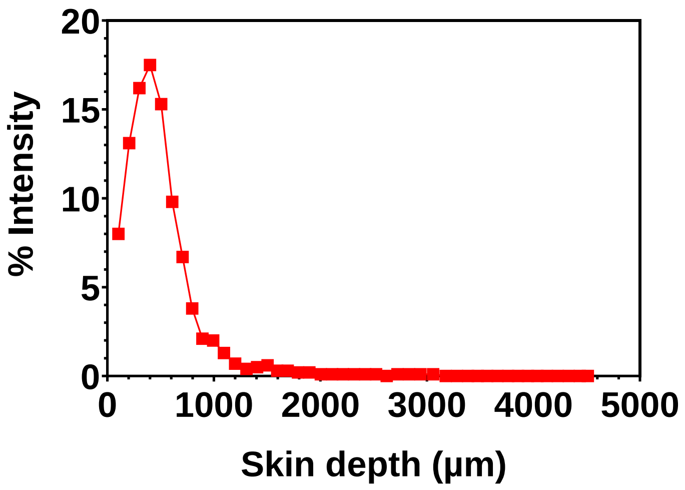

Figure S12.2. Permeation plot of TBF to human abdominal skin using the ethosomal DDS, calculated using the python code.

S13. Control with TBF in TDW

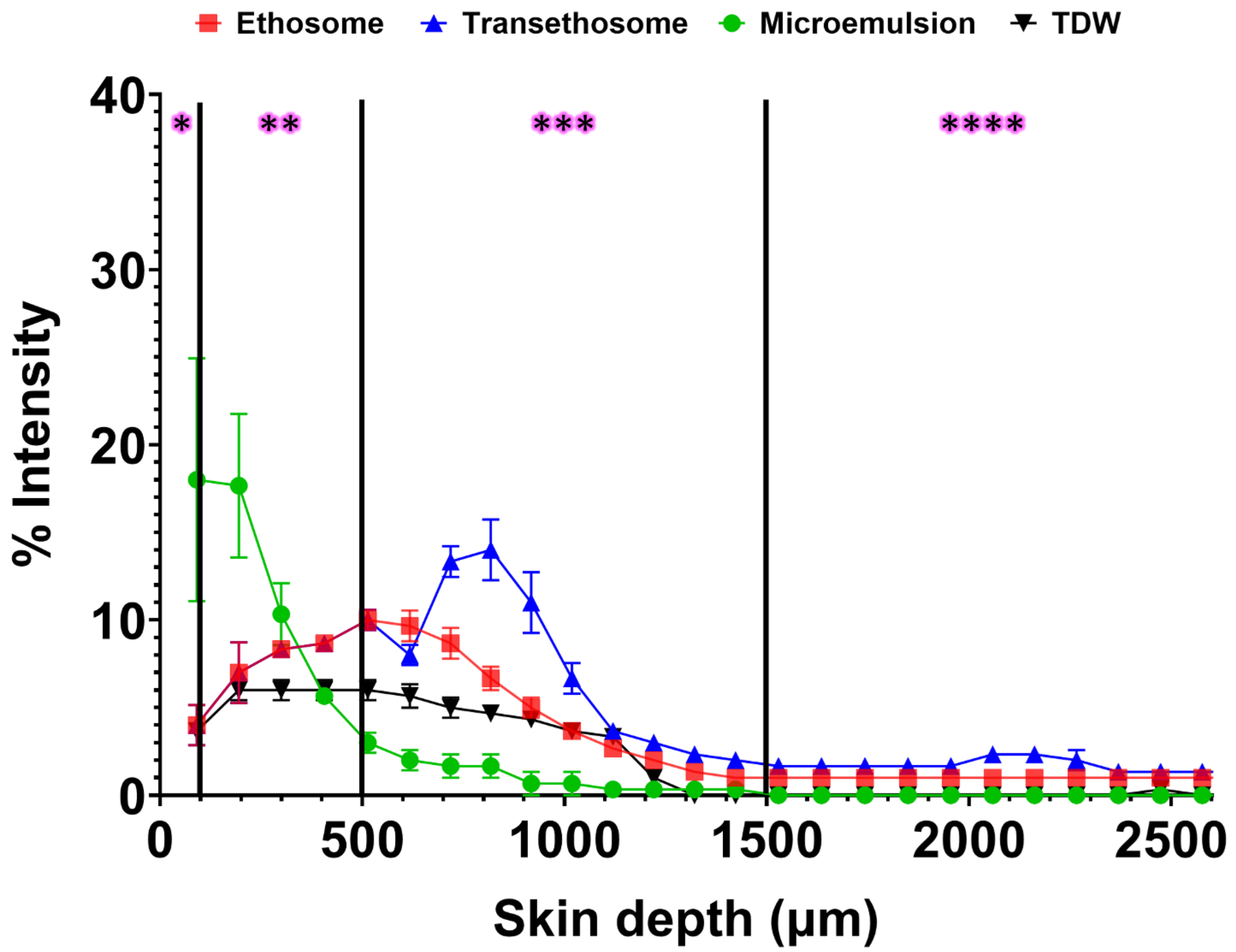

Figure S13. TBF permeation to human abdominal skin after 2 hours of application. Comparison between different DDSs and a control of TDW. *-epidermis, **-upper dermis, ***-lower dermis, ****-hypodermis.

S14. Comparison between FITC and TBF permeation to skin

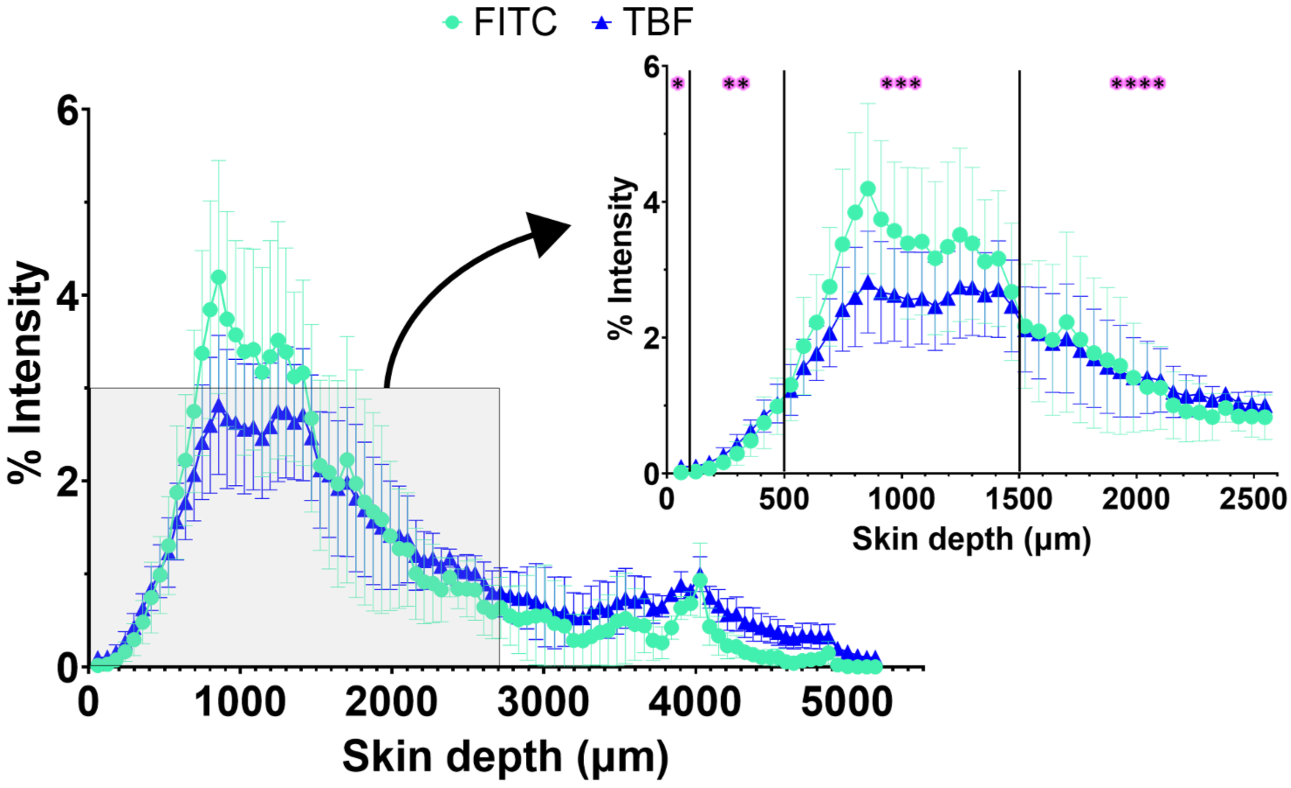

Figure S14. TBF and FITC permeation to human abdominal skin from transethosome DDS. *-epidermis, **-upper dermis, ***-lower dermis, ****-hypodermis.
